# Edge-Deployable Lightweight Deep Learning for Hypertensive Retinopathy Grading from En-Face OCT: A Patient-Level Feasibility Study

**DOI:** 10.3390/bioengineering13090984

**Published:** 2026-08-26

**Authors:** Süleyman Burçin Şüyun, Mustafa Yurdakul, Şakir Taşdemir, Serkan Biliş

**Affiliations:** 1 Digital Transformation Office, The Rectorate of Sinop University, Sinop University, 57000 Sinop, Türkiye; 2 Computer Engineering Department, Kırıkkale University, 71450 Kırıkkale, Türkiye; 3 Computer Engineering Department, Selçuk University, 42250 Konya, Türkiye; 4 Eye Diseases Department, Batıgöz Medical Group Hospital, 35200 İzmir, Türkiye

**Keywords:** hypertensive retinopathy, lightweight deep learning, edge AI, en-face OCT image classification, edge deployment, patient-level cross-validation, Grad-CAM, Keith–Wagener–Barker, point-of-care triage

## Abstract

**Background:** Hypertensive retinopathy (HR) is an early marker of hypertension-related end-organ damage, and multi-grade classification is limited by overlap between the early stages. No prior study has reported edge-deployable HR grading from optical coherence tomography (OCT). We assess patient-level–validated four-grade HR classification from en-face OCT with on-device inference. **Methods:** MobileViT-XXS and EfficientNetV2-B0 were evaluated on 478 en-face OCT images from 221 patients. To avoid leakage, all partitions were patient-level: stratified group 5-fold cross-validation with a patient-disjoint test set. Each model was evaluated as a 5-fold soft-voting ensemble, with inference profiled on an NVIDIA Jetson Orin Nano. Accuracy, weighted F1, quadratic-weighted kappa, and ROC–AUC were reported; the models were compared by McNemar’s test, and clinical utility by referable-HR (Grade ≥ 2) triage. **Results:** MobileViT-XXS achieved 84.4% accuracy, ROC–AUC 0.944, and quadratic-weighted kappa 0.916, significantly outperforming EfficientNetV2-B0 (McNemar *p* = 0.013). Mild-grade overlap limited four-class accuracy, but referable-HR triage reached 95.6% accuracy, 95.2% sensitivity, and 95.8% negative predictive value. MobileViT-XXS required only 3.83 MB versus 23.70 MB, and on-device TensorRT FP16 five-fold ensemble inference achieved 10.4 ms (96 FPS) at 8.9 W. **Conclusions:** Lightweight models enable feasible, power-efficient point-of-care HR triage from en-face OCT; broader multi-center validation is warranted.

## 1. Introduction

Hypertension is one of the most prevalent cardiovascular conditions, affecting approximately 1.3 billion adults aged 30–79 years worldwide [1]. Shifts in lifestyle, rising obesity, and the aging and growth of the world’s population have increased the number of people with high blood pressure in both developed and developing countries [2]. Untreated high blood pressure damages several organ systems, and because of its direct relationship with the vascular system, the retina is among the first organs affected. The retinal vasculature is consequently an informative marker of the circulatory changes produced by hypertension. Vessel narrowing, arteriovenous crossing changes, damage to the blood–retinal barrier, exudates, and hemorrhages are the characteristic retinal findings of hypertensive retinopathy (HR) [3]. HR is clinically staged with the Keith–Wagener–Barker (KWB) framework [4]. Later clinical reviews simplified that framework while preserving its progression, from no findings (Grade 0) to severe manifestations including retinal hemorrhages, exudates, and optic disc edema (Grade 3) [5].

Early detection of hypertensive retinopathy is important for preventing vision loss and limiting hypertension-related comorbidities. Fundus photography is the established modality for identifying early HR signs, and non-mydriatic cameras combined with semi-automated vessel-analysis software have expanded its use from hospital to large-scale screening settings [6]. However, variation in image quality, color, and brightness across devices continues to limit automated detection.

Over the past decade, automated retinal disease diagnosis from fundus images has been dominated by deep CNNs such as ResNet, DenseNet, Inception, and EfficientNet, which achieved strong results for diabetic retinopathy, glaucoma, and related conditions [7]. Convolutional models have similarly been applied to retinal vascular disease classification [8]. Comparative studies in other radiographic domains suggest that the choice of backbone is a smaller determinant of performance than dataset composition and evaluation protocol, a conclusion reached directly by a systematic comparison of five CNN architectures for osteoporosis prediction from the mandibular cortical index on panoramic radiographs [9]. For hypertensive retinopathy specifically, early CNN systems outperformed traditional machine-learning pipelines [10]. These were later refined by CNN–SVM hybrids [11]. Most recently, a three-stage approach combining CNN features, machine-learning classifiers, and metaheuristic optimization reported the highest accuracy on a single train–test split [12]. These methods, however, typically rely on computationally intensive architectures and single-split evaluation, and are developed almost exclusively for fundus photography. In parallel, transformer and lightweight CNN–Transformer hybrids such as MobileViT have gained attention for modeling global context with few parameters [13]. Related work includes retinal vessel segmentation as a route to the same global information [14]. Hybrid Vision Transformer models for four-grade HR grading have also been reported to outperform standalone CNNs [15]. Lightweight vision transformers have since been applied to ordinal staging of other ophthalmic diseases, including early glaucoma grading from fundus images [16].

Despite this progress, no lightweight, edge-deployable HR grading system has been reported. This gap matters: early retinal changes offer a direct, non-invasive window into systemic vascular health [3], yet the lack of a deployable tool limits point-of-care use where specialist access is scarce. Prior work also shares recurring weaknesses. Datasets are typically small for a condition of this prevalence [8]. Inter-observer variability is high for the early grades, and automated confusion between adjacent grades follows from the same visual overlap [17]. The limited diagnostic yield of routine funduscopy in mild-to-moderate hypertension has been documented for the same reason [18]. Class imbalance, camera-induced quality variation, and single-split evaluation compound these constraints on generalization [19]. Computer-aided diagnostic reviews of hypertensive retinopathy report the same set of limitations [20]. The difficulty of ordinal severity grading is not confined to ophthalmology: automated staging of periodontal bone loss on bite-wing radiographs shows the same pattern of strong performance at the extremes of the scale and weaker separation of adjacent intermediate stages [21]. Unlike the accuracy-oriented three-stage GPU pipeline previously reported for this cohort [12], the present work prioritizes lightweight, edge-deployable models, accepting a moderate accuracy trade-off in exchange for near-real-time on-device operation. Recent studies continue to advance AI-based retinal analysis for hypertension-related disease, for example multimodal fundus–OCT frameworks for systemic vascular risk stratification [22]; on-device HR grading from OCT, however, remains unaddressed.

Whereas most HR studies use fundus photography, the present study uses retinal en-face OCT images acquired during routine screening. En-face OCT provides complementary structural information on the retinal vasculature and is increasingly used to study hypertension-related changes. These images share substantial visual overlap with fundus photographs in the retinal structures relevant to vascular assessment, and the KWB-inspired criteria—originally defined for fundus examination—were applied to them by a board-certified ophthalmologist, supporting the use of deep-learning architectures developed for fundus classification on this OCT data.

This study addresses three key gaps in the literature: (i) the limited investigation of lightweight, end-to-end compatible CNN–Transformer hybrid architectures for hypertensive retinopathy classification, (ii) insufficient utilization of multi-fold evaluation methods to enhance generalizability, and (iii) the persistent challenge of discriminating between the clinically similar Grade 1 and Grade 2 stages. The main contributions of this study are as follows:

- Development and comparative evaluation of a lightweight MobileViT-XXS CNN–Transformer hybrid and EfficientNetV2-B0 under identical experimental settings for four-grade HR classification.
- Robust performance assessment using stratified 5-fold cross-validation combined with an independent test set, providing reproducible internal evaluation beyond single train–test splits.
- Systematic investigation of the challenging Grade 1–Grade 2 discrimination problem through class-wise precision, recall, and confusion matrix analysis across all cross-validation folds.
- Near-real-time 5-fold ensemble deployment and validation on the NVIDIA Jetson Orin Nano Developer Kit via a Gradio-based web interface, demonstrating the technical feasibility of on-device inference toward potential point-of-care HR screening in resource-limited settings, where access to specialist screening is scarce [23], pending external clinical validation.

Although each of these components has individual precedents, the primary novelty of this work lies in their integration: to our knowledge, four-grade hypertensive retinopathy grading from en-face OCT images using lightweight convolutional and CNN–Transformer architectures with validated on-device inference has not previously been reported as a single, edge-deployable pipeline.

Section 2 describes the dataset, preprocessing procedures, model architectures, and experimental configurations. Section 3 presents 5-fold cross-validation and independent test results, comparing them with other published studies. Section 4 interprets the findings and evaluates the study’s limitations. Section 5 concludes the work and offers directions for future research.

## 2. Materials and Methods

### 2.1. Dataset

**Figure 1:**
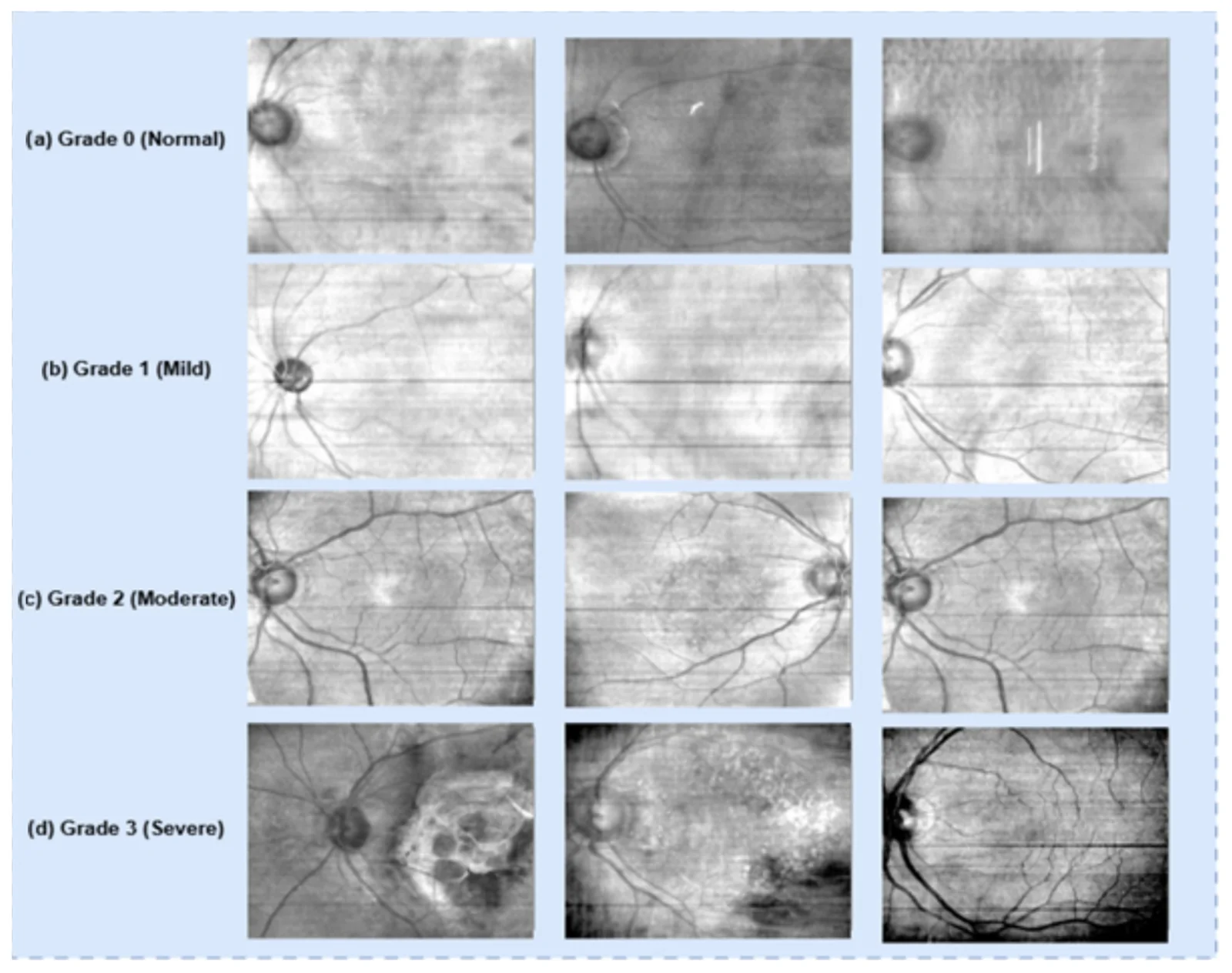
Representative en-face OCT images from the dataset across four hypertensive retinopathy grades based on a KWB-inspired–grading scheme. (**a**) Grade 0—Normal, (**b**) Grade 1—Mild, (**c**) Grade 2—Moderate, (**d**) Grade 3—Severe.

The en-face OCT images used here were acquired at the same institution and under the same acquisition protocol as the image-level dataset previously reported by Şüyun et al. [12], which used a “Normal/Stage 1–3” severity terminology; the present study adopts an equivalent “Grade 0–3” nomenclature aligned with the KWB framework, without any change to the underlying class definitions. The dataset consists of en-face OCT images collected from patients in Türkiye who underwent routine ophthalmologic screening examinations. These en-face OCT images were derived from macular volumetric (cube) scans acquired with a spectral-domain OCT device. Images from both eyes of each subject were acquired, and no distinction between right and left eyes was used during model development, as hypertensive retinopathy grading under this KWB-inspired scheme is based on the severity of retinal findings irrespective of laterality. The dataset consisted of four classes corresponding to the clinically identifiable stages of hypertensive retinopathy (Grade 0–3). All images were manually annotated using a grading scheme adapted from the Keith–Wagener–Barker classification by a board-certified ophthalmologist with extensive clinical experience in retinal diseases. In cases of uncertainty, images were re-evaluated in a separate review session to ensure labeling consistency. Representative en-face OCT images for each grade are shown in Figure 1.

Table 1 presents the class-wise image distribution for the cross-validation pool, while Table 2 shows the class-wise image distribution for the independent test set. From these same-source en-face OCT acquisitions, the present study assembles a patient-level, quality-controlled dataset for leakage-free evaluation. Because all images were fully anonymized for the present study, individual patient- or image-level correspondence with the earlier dataset of Şüyun et al. [12] cannot be established; consequently, partial overlap with that dataset can be neither confirmed nor excluded, and no claim of dataset independence is made. The two datasets share the same institution and acquisition protocol but differ in curation and organization: whereas [12] was partitioned image-wise without patient grouping, the present study links each image to an anonymized sequential patient identifier and eye laterality (OD/OS) and excludes patients whose images were of insufficient quality, yielding a patient-level dataset (hereafter HRGrade-OCT-PL) of 478 unique images from 221 patients (336 eyes; 240 right-eye [OD] and 238 left-eye [OS] images). All retained images were verified to be unique by content hashing and of uniform resolution. Of these, 388 images (from 176 patients) were used for stratified group five-fold cross-validation, and 90 images (from 45 patients) formed a patient-disjoint, held-out internal test set drawn from the same institution and acquisition protocol; this internal test set does not constitute external validation. Crucially, all partitioning was performed at the patient (subject) level so that no patient contributed images to both the training and test sets, thereby eliminating subject-level data leakage. The training and test subsets were stratified across all four clinical classes.

To ensure an unambiguous grading scheme, each grade was defined by explicit clinical criteria adapted from the Keith–Wagener–Barker (KWB) framework, and its correspondence to the original KWB groups is stated explicitly as follows. Grade 0 (normal) denotes no hypertension-related retinal changes; it is newly introduced in this adapted scheme and has no counterpart in the original KWB system, which does not include a dedicated normal category. Grade 1 corresponds to KWB Group I (mild generalized arteriolar narrowing and vessel-wall thickening). Grade 2 corresponds to KWB Group II (more pronounced focal vasoconstriction with arteriovenous crossing changes and arteriosclerotic features). Grade 3 corresponds to KWB Group III (advanced vascular involvement with retinal hemorrhages, hard exudates, and cotton-wool spots); the rare KWB Group IV (malignant hypertensive retinopathy with optic-disc edema/papilledema) was not represented as a separate class and, when present, was collapsed into Grade 3. The scheme is therefore KWB-inspired rather than a one-to-one reproduction of the original four-group system. All images were graded against these criteria by a board-certified ophthalmologist (co-author) with extensive retinal experience, with images of uncertain grade re-evaluated in a separate session to ensure labeling consistency.

The study protocol was approved by the Institutional Ethics Committee of Selçuk University (Approval No: 2025/281), and all images were anonymized prior to analysis.

### 2.2. Experimental Setup and Validation Strategy

The variability of simple training and test set splitting in medical images has been widely reported [24]. The dependence of a reported performance estimate on the particular train/test partition has been quantified directly [25]. To address this and to prevent subject-level data leakage, stratified group five-fold cross-validation was applied at the patient level. First, a patient-disjoint test set of 90 images (45 patients) was set aside and kept completely independent of all training and tuning procedures. The remaining 388 images (176 patients) were then partitioned into five folds using stratified group k-fold, grouping by patient so that all images from a given patient remained within a single fold and no patient appeared in more than one fold. In each iteration, one fold was used for validation, and the other four for training, and every image was validated exactly once. We verified that the resulting partitions contained no patient overlap between training and test (zero leakage), that each eye carried a single grade across all folds, and that all images were unique and of uniform resolution. The results reported here include the mean metrics across the five folds with standard deviations, together with a 5-fold soft-voting ensemble evaluated on the independent test set.

### 2.3. Preprocessing and Data Augmentation

In this study, two different deep-learning architectures, EfficientNetV2-B0 and MobileViT-XXS, were employed, and for both models, the architectural requirements suggested in the literature for en-face OCT images were considered. However, preprocessing procedures were performed separately, taking best practices into account. All en-face OCT images were resized to each model’s input size (256 × 256 pixels for MobileViT-XXS and 224 × 224 pixels for EfficientNetV2-B0) to ensure compatibility with the input layers of the models. To prevent leakage of preprocessing statistics between the training, validation, and test sets, only basic normalization was applied in the validation and testing phases.

#### 2.3.1. Input Normalization

All en-face OCT images were first resized to each model’s input size (256 × 256 pixels for MobileViT-XXS and 224 × 224 pixels for EfficientNetV2-B0). Input normalization was then applied in a model-specific manner: EfficientNetV2-B0 used its dedicated preprocess_input routine, which reproduces the normalization scheme under which the network was pretrained on ImageNet, whereas MobileViT-XXS used simple rescaling of pixel intensities to the [0, 1] range by a factor of 1/255. This difference is intentional: each backbone was pretrained on ImageNet under its own input-normalization convention, and supplying inputs normalized in the way the pretrained weights expect preserves the validity of the transferred features and stabilizes fine-tuning. Using the EfficientNetV2-B0 preprocess_input for that model and the reference [0, 1] scaling for MobileViT-XXS therefore matches each network to its respective pretraining distribution, rather than imposing a single normalization that would be mismatched for one of the two backbones.

#### 2.3.2. Data Augmentation

Data augmentation was applied identically to both architectures and only to the training set; validation and test images received the model-specific normalization described above without any augmentation. Because the two training pipelines share the same augmentation configuration, it is described here once rather than repeated for each model. The augmentation comprised random rotation within −20° to +20°, horizontal and vertical translation of up to 10% of the corresponding image dimension, shear of up to 10%, scaling within the 0.85–1.15 range, and random brightness adjustment within the [0.8, 1.2] range. Horizontal flipping was used while vertical flipping was not, and regions exposed by the geometric transformations were filled by reflection padding. These operations improve generalization to variability in acquisition conditions and inter-subject anatomical differences without altering the underlying grade of the image.

#### 2.3.3. Excluded Operations

For both models, advanced image enhancement techniques such as CLAHE, sharpening, blurring, and histogram equalization were not applied. Models were trained and evaluated using original en-face OCT images.

### 2.4. Model Architectures

Two edge-oriented architectures were compared for hypertensive retinopathy classification: MobileViT-XXS, a lightweight CNN–Transformer hybrid, and EfficientNetV2-B0, a compact CNN—both selected for their low computational cost and portability to mobile and edge systems.

#### 2.4.1. MobileViT-XXS Architecture

MobileViT-XXS is a lightweight CNN–Transformer hybrid architecture combining convolutional feature extraction with global attention mechanisms [13]. The XXS variant represents the smallest configuration of the MobileViT family and was selected for edge device deployment.

**Figure 2:**
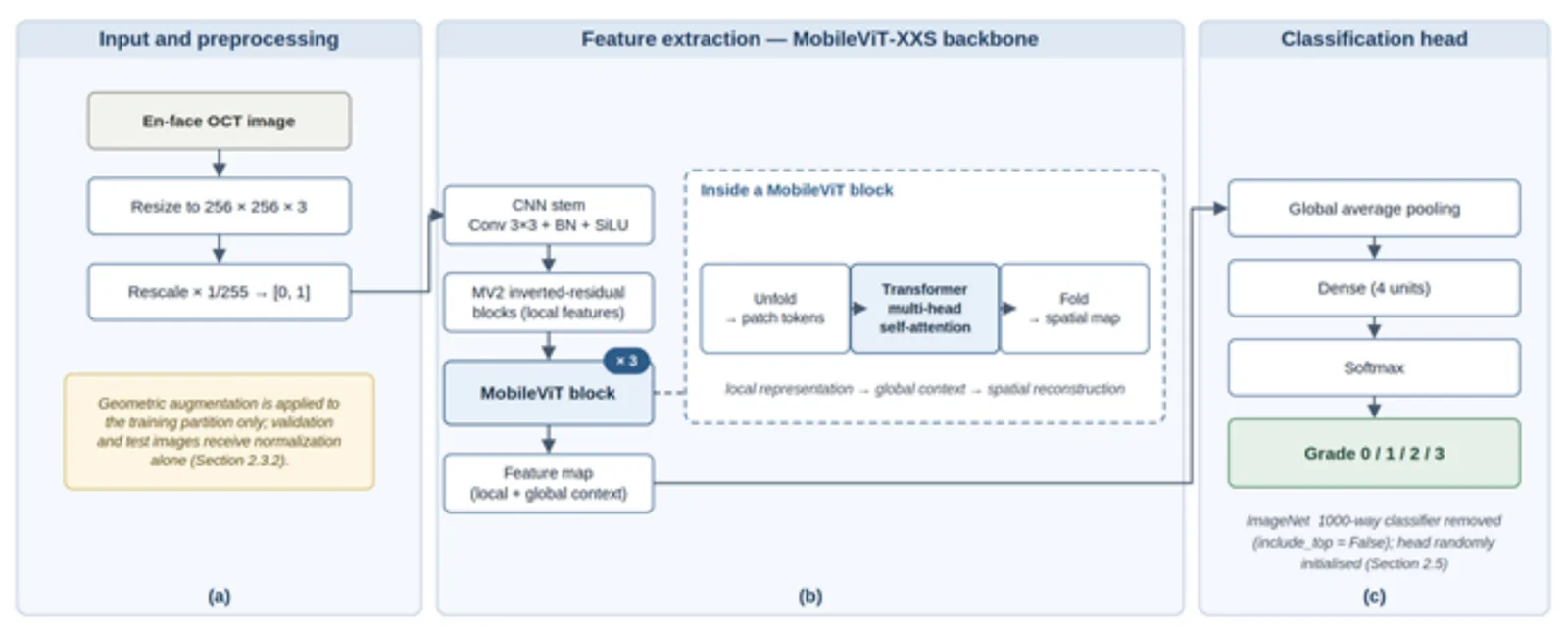
MobileViT-XXS-based hypertensive retinopathy classification architecture. (**a**) Input and preprocessing: the en-face OCT image is resized to 256 × 256 × 3 and rescaled to [0, 1]; (**b**) feature extraction with the MobileViT-XXS backbone, comprising a CNN stem, MV2 inverted-residual blocks and three MobileViT blocks that combine local convolution with transformer multi-head self-attention; (**c**) classification head, with global average pooling, a dense layer (4 units) and a softmax producing the Grade 0–3 prediction.

As illustrated in Figure 2, the MobileViT-XXS framework comprises (a) input preprocessing, (b) MobileViT-block feature extraction, and (c) classification. Inputs are resized to 256 × 256 and scaled to [0, 1]. A convolutional stem consisting of a 3 × 3 convolution, batch normalization, and a non-linear activation extracts low-level features such as retinal vasculature and texture. Each MobileViT block (Figure 2b) then adds global context in three steps: the local feature map is unfolded into fixed-size patch tokens, those tokens are processed by a transformer block with multi-head scaled dot-product self-attention, and the result is folded back onto the original spatial grid, yielding a representation that combines local and global context. The block formulations are those of the original architecture and are not reproduced here; the backbone was used unmodified [13]. The classification head (Figure 2c) applies global average pooling followed by a dense softmax layer that outputs the probability distribution over the four hypertensive retinopathy grades.

#### 2.4.2. EfficientNetV2-B0 Architecture

EfficientNetV2-B0 was included as a widely used CNN baseline in medical imaging. The EfficientNetV2 family offers faster convergence and a balance between accuracy and computation costs compared to the previously developed EfficientNet series [26].

**Figure 3:**
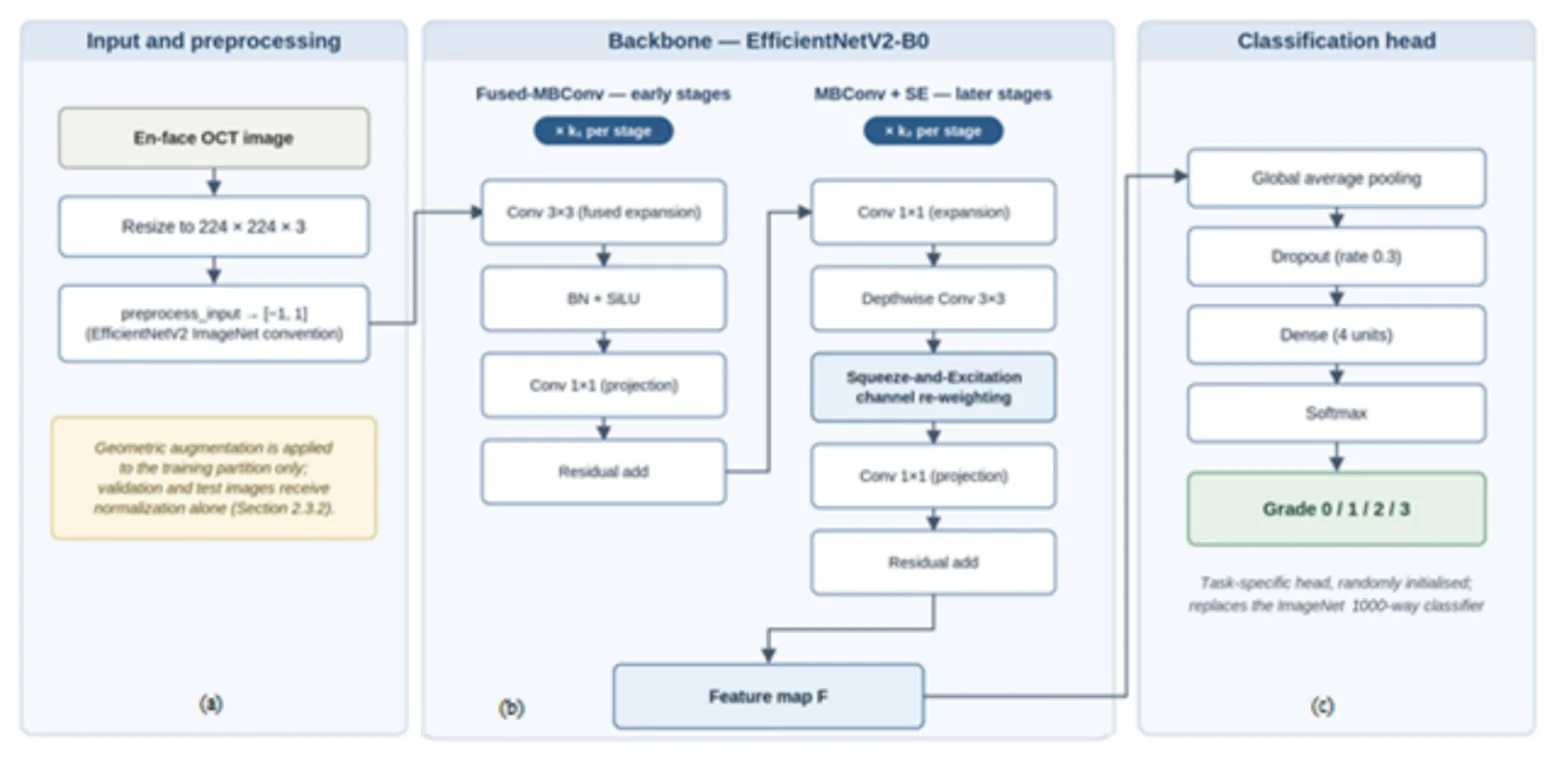
EfficientNetV2-B0-based hypertensive retinopathy classification architecture. (**a**) Input and preprocessing: the en-face OCT image is resized to 224 × 224 × 3 and normalized with the EfficientNetV2 ImageNet convention to [−1, 1]; (**b**) EfficientNetV2-B0 backbone, with Fused-MBConv blocks in the early stages and MBConv blocks with squeeze-and-excitation in the later stages, producing feature map F; (**c**) classification head, with global average pooling, dropout (0.3), a dense layer (4 units) and a softmax producing the Grade 0–3 prediction.

As shown in Figure 3, the EfficientNetV2-B0 pipeline comprises (a) input preprocessing, (b) backbone feature extraction, and (c) classification. En-face OCT images are resized to 224 × 224 and normalized with the architecture’s own preprocess_input function, which maps intensities to the [−1, 1] range expected by the released ImageNet weights; the augmentation policy applied to the training partition is specified once in Section 2.3.2. The backbone uses Fused-MBConv blocks in its early stages, where a single 3 × 3 convolution replaces the separate expansion and depth-wise convolutions and lowers computational cost, and MBConv blocks with Squeeze-and-Excitation channel attention in the later stages, where global average pooling produces channel descriptors that are passed through a bottleneck and a sigmoid gate to re-weight the feature channels (Figure 3b). Block groups are repeated according to the standard EfficientNetV2-B0 configuration, and the standard block formulations are given in the original description of the architecture rather than reproduced here [26]. The classification head (Figure 3c) applies global average pooling to the final backbone feature map, followed by a dropout layer (rate 0.3) and a dense layer with four units and softmax activation, producing the probability distribution over the four hypertensive retinopathy grades. Efficiency-oriented designs that pair MBConv blocks with lightweight channel attention have also been adopted in compact detection frameworks for other medical imaging tasks [27].

To enable a fair architectural comparison, the EfficientNetV2-B0 model was trained end-to-end in a single stage using the same optimization protocol as MobileViT-XXS, without any layer freezing or staged fine-tuning. Both models were subsequently deployed on an NVIDIA Jetson Orin Nano Developer Kit for near-real-time inference evaluation, as detailed in Section 2.7.

### 2.5. Training Setup

Both architectures were trained on identical patient-level folds under a single, common protocol, so that any difference in performance is attributable to the backbone rather than to the training strategy. The settings are stated once here and are not repeated elsewhere.

Inputs were presented at 256 × 256 pixels for MobileViT-XXS and 224 × 224 pixels for EfficientNetV2-B0, matching each architecture’s native input resolution. The batch size was 16, the epoch budget was 50 for both models, and the augmentation configuration was identical and is described once in Section 2.3.2. Each of the five folds was initialized and optimized independently, producing five models per architecture; the 90-image, patient-disjoint test set was held back throughout.

Both models were initialized from ImageNet-pretrained weights, with the original 1000-class ImageNet classifier discarded (include_top = False) and the pretrained backbone retained as a feature extractor. MobileViT-XXS received global average pooling followed by a four-unit softmax layer; EfficientNetV2-B0 received global average pooling, a dropout layer (rate 0.3), and a four-unit softmax layer. Both heads were randomly initialized, and both models were trained end-to-end in a single stage with no layer freezing, so that backbone and head parameters were updated jointly.

Both architectures used categorical focal loss (γ = 2.0) with an α vector obtained by normalizing the balanced class weights returned by compute_class_weight, and both were optimized with Adam at an initial learning rate of 1 × 10^−4^. Because every layer starts from pretrained weights and is updated jointly in a single end-to-end stage, a uniformly low learning rate was used so that all layers receive small, stable updates that preserve the transferred features. Early stopping on the validation loss (patience = 10) with restoration of the best-validation weights was applied to both models, together with a ReduceLROnPlateau schedule (factor = 0.5, patience = 4). The model retained for each fold therefore corresponds to the epoch with the lowest validation loss rather than to the final epoch, which directly mitigates the train–validation gap discussed in Section 3.1. Trained models were saved in the Keras native format for on-device deployment (Section 2.7).

#### Hardware and Software Environment

All training and evaluation processes were carried out on a dedicated hardware and software platform. The computations were carried out using two NVIDIA RTX A5500 graphics cards, each having 24 GB of memory. The environment was set up to support CUDA version 12.x, with Python version 3.11 and TensorFlow Keras, a deep-learning framework. Each fold model was trained as an independent single-GPU job on this dual-GPU platform. To support reproducibility, the stratified train/test split and the five-fold partitioning were performed at the patient level using a stratified group k-fold procedure (grouping by patient). The resulting fold assignments are provided in the code repository so that all experiments can be repeated on identical splits. Model weight initialization and mini-batch shuffling during training were not seed-fixed; accordingly, minor run-to-run variation is expected in individual training runs, and all reported metrics are therefore presented as the mean ± standard deviation across the five folds.

During manuscript preparation, a generative AI assistant (Claude, Anthropic) was used for language editing and for drafting and revising manuscript text, including passages of the Discussion. It was not used for study design, data collection, image labeling, model training, statistical computation, or the generation of any experimental result; every numerical value, figure and table reported here was produced by the authors’ own code and hardware. All scientific claims, interpretations and conclusions were formulated, verified against the underlying data and approved by the authors, who take full responsibility for the content of this publication.

### 2.6. Evaluation Metrics and Statistical Analysis

Model performance was assessed using a set of complementary metrics for multi-class medical image classification, combining general accuracy measures with statistics that account for class imbalance and chance agreement:

- Overall accuracy: The proportion of correctly classified instances out of all instances, providing a general measure of classification performance.
- Precision (weighted): The proportion of true positive predictions among all positive predictions, weighted by class frequency to account for class imbalance.
- Recall (weighted): The proportion of true positive instances correctly identified among all actual positives, weighted by class frequency. This metric is of particular importance in clinical classification tasks where missed diagnoses carry significant consequences.
- F1-score (weighted average): This is a weighted average of precision and recall computed as a harmonic mean to ensure a balanced assessment of model performance over imbalanced datasets.
- Cohen’s Kappa (unweighted): This is a quantification of the agreement between predictions made by the model and ground truth labels. This was used to evaluate the true potential of the model.
- ROC–AUC (one-vs-rest, multi-class): To evaluate the class discrimination capability of the model, the one-vs-rest (OvR) strategy was used in a multi-class setup. In this setup, each class is compared to all other classes individually. The computed AUC values are reported individually for each class as well as macro and weighted averages.
- Confusion matrix (raw counts): To evaluate the relationship between actual and predicted class labels in more detail, a raw count-based confusion matrix was computed for each fold.

All the above-mentioned evaluation metrics have been computed using the scikit-learn library. To calculate ROC–AUC for the multi-class setting, the OvR strategy was used. Performance is reported individually for each fold and is compared by computing mean values to understand the behavior of the models.

To determine whether the performance differences between the two architectures were statistically meaningful rather than attributable to random variation, a paired statistical comparison was performed. Because both models were trained under identical five-fold cross-validation splits and each fold model was evaluated on the same fixed 90-image independent test set, the five per-fold values were treated as paired observations. For accuracy, balanced accuracy, macro-averaged F1, and quadratic-weighted kappa, EfficientNetV2-B0 and MobileViT-XXS were compared using a two-sided paired *t*-test, with the non-parametric Wilcoxon signed-rank test additionally reported. Given the small sample (*n* = 5 folds), the paired *t*-test was treated as the primary fold-wise test (in preference to Wilcoxon), since the two-sided Wilcoxon signed-rank test cannot yield a *p*-value below 0.0625 when all five paired differences share the same sign (and, for accuracy, no lower than 0.125, since one fold is tied and its zero difference is dropped, leaving four). Statistical significance was set at α = 0.05. Because all five fold models were evaluated on the same fixed test set, these per-fold values are not statistically independent; the fold-wise paired test is therefore exploratory and reported as descriptive. The primary, statistically valid architecture comparison is the sample-level McNemar test, which contrasts the two ensembles on identical test instances.

### 2.7. Edge Device Deployment

**Figure 4:**
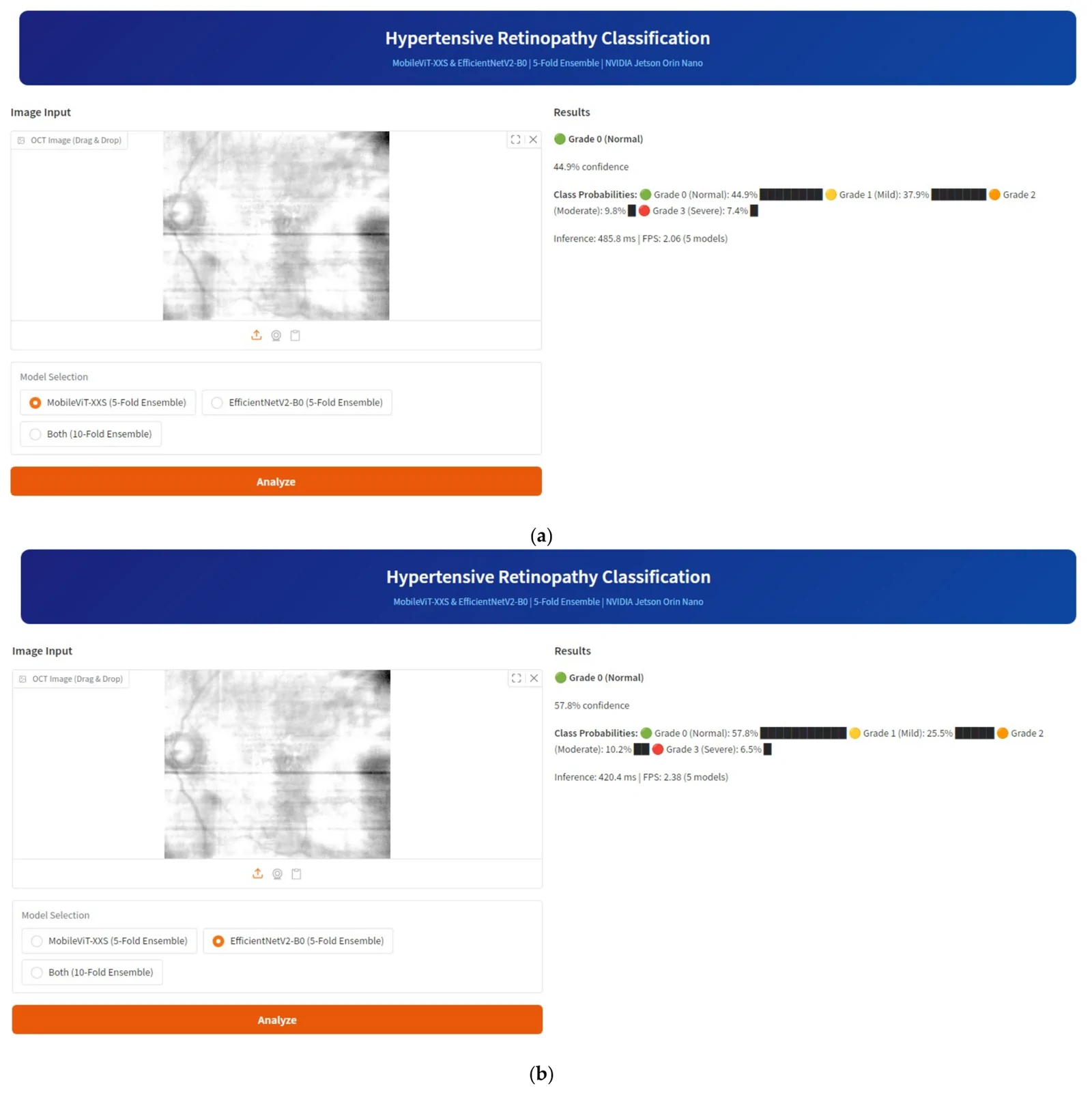
Gradio-based web interface deployed on the NVIDIA Jetson Orin Nano Developer Kit, showing on-device ensemble inference results. (**a**) MobileViT-XXS 5-fold ensemble: 485.8 ms warm-start inference time, Grade 0 (Normal) prediction with 44.9% confidence. (**b**) EfficientNetV2-B0 5-fold ensemble: 420.4 ms warm-start inference time, Grade 0 (Normal) prediction with 57.8% confidence.

Both models were deployed on an NVIDIA Jetson Orin Nano Developer Kit equipped with a 1024-core Ampere GPU, 8 GB unified memory, and running JetPack 6.2.1 with TensorFlow 2.16.1. A Gradio-based web interface was developed to enable drag-and-drop en-face OCT image upload and near-real-time ensemble inference directly on the edge device, as illustrated in Figure 4.

Following training, all fold models were saved in the Keras native format (.keras). These .keras files are made available in the code repository alongside the training scripts to support full reproducibility of the training pipeline. Prior to deployment, both model families were exported to the TensorFlow SavedModel format for compatibility with TensorFlow 2.16.1 on the edge device. The .keras files were loaded with the tf_keras (Keras 2-compatible) API—which natively resolves the layer configurations of both architectures, including the TFOpLambda layers of MobileViT-XXS, without additional configuration patching—and re-saved as self-contained SavedModels. The complete conversion scripts are provided in the code repository, and the resulting SavedModels are fully compatible with TensorFlow 2.16.1 on the Jetson Orin Nano Developer Kit; these SavedModels serve as the common starting point for both the FP32 Gradio interface and the TensorRT FP16 optimization pipeline (SavedModel → ONNX opset 13 → TensorRT FP16 engine).

The inference tests were conducted on the Jetson Orin Nano Developer Kit. The en-face OCT images were preprocessed and normalized according to the requirements of the models: EfficientNetV2-B0 used the preprocess_input function, normalizing to the [−1, 1] range, while MobileViT-XXS used 1/255 scaling to the [0, 1] range. Inference was performed at FP32 precision using TensorFlow 2.16.1. The first inference required GPU warm-up time of approximately 70 s, while subsequent inferences achieved stable latency. MobileViT-XXS has a total of 956,420 parameters (952,308 trainable) with a model size of 3.83 MB, and achieved an average single-model inference time of 102.4 ± 22.7 ms at 9.77 FPS. EfficientNetV2-B0 has a total of 5,924,436 parameters (5,863,828 trainable) with a model size of 23.70 MB, and achieved an average single-model inference time of 80.7 ± 15.7 ms at 12.39 FPS. After TensorRT FP16 optimization, each per-fold engine occupied 6.0 MB (MobileViT-XXS) and 13.9 MB (EfficientNetV2-B0) on-device. TensorRT FP16 optimization enabled near-real-time inference on the edge device for both models, confirming their suitability for deployment in resource-limited clinical settings.

### 2.8. Grad-CAM Computation

Post-hoc interpretability was assessed with gradient-weighted class activation mapping (Grad-CAM). For each architecture, the gradients of the target class score with respect to the activations of a selected convolutional feature map were global-average-pooled to obtain per-channel weights; the weighted sum of the activation channels was passed through a ReLU, normalized to the unit interval, resampled to the model input resolution and overlaid on the source en-face OCT image using a jet color map, so that red denotes the highest normalized activation within each individual map. The target layer was the final Conv2D layer for EfficientNetV2-B0 and, for MobileViT-XXS, the final convolutional feature map of the network (the output projection after the last MobileViT block), because the folded patch-token representation produced inside a MobileViT block does not expose a spatially aligned convolutional activation tensor to which Grad-CAM can be applied directly.

Two properties of this procedure bound what the resulting maps can show and are stated here so that they are not read as limitations discovered after the fact. First, Grad-CAM inherits the spatial granularity of the layer at which it is computed: at a 224 × 224 input the final convolutional stage of EfficientNetV2-B0 is a 7 × 7 grid, so a single activation cell already corresponds to a 32 × 32-pixel block—roughly one-seventh of the field of view in each direction—and the apparent spatial spread of a map is therefore partly an artefact of up-sampling a very coarse grid. Second, the maps were generated from the fold-1 model of each architecture and not from the five-fold soft-voting ensemble, for which single-model gradient attribution is not strictly defined; they are accordingly illustrative of one ensemble member rather than an explanation of the deployed system.

The dataset used here carries image-level KWB-inspired grades only and contains no pixel-wise or bounding-box annotation of individual hypertensive retinopathy findings. No overlap statistic between the attention maps and a lesion reference (for example, intersection-over-union, Dice, or pointing-game accuracy) could therefore be computed, and the Grad-CAM analysis reported in Section 4.7 is explicitly a qualitative appraisal of attention–pathology concordance rather than a validated localization result.

For the case-level analysis in Section 4.7, representative successful and failure cases were selected after computing Grad-CAM maps with the fold-1 models using the procedure described above. All annotations on the source images were placed by the ophthalmologist co-author directly on the images—yellow arrows marking visible structural findings and pink arrows marking regions that attract strong model attention without containing strongly discriminative hypertensive retinopathy findings; these indications are qualitative and do not constitute pixel-level lesion annotations.

## 3. Results

### 3.1. Training Performance

The training and validation curves for all five cross-validation folds are provided in the code repository. Both models were trained end-to-end under an identical protocol (focal loss with balanced class weights, the Adam optimizer at 1 × 10^−4^, and early stopping on the validation loss), so that differences in their learning dynamics reflect the architectures rather than the training strategy. In both models, the training accuracy rises well above the validation accuracy, indicating a degree of overfitting expected on a dataset of this size; early stopping with restoration of the best-validation weights mitigates this by retaining the best-generalizing checkpoint for each fold.

For MobileViT-XXS (training curves in the code repository), training loss decreases consistently across all folds, reaching approximately 0.03–0.06 at the early-stopping point (all folds terminated between epochs 12 and 28 of the 50-epoch budget). However, validation loss plateaus at around 0.11–0.15 without a corresponding downward trend, and validation accuracy plateaus in the approximate range of 0.50–0.62 across folds, indicating a train–validation gap attributable to the model’s capacity relative to the limited dataset size. Among the folds, Folds 1 and 4 exhibit the most pronounced validation loss variability, likely reflecting distribution differences within those data partitions. The application of focal loss with class weights and data augmentation partially mitigates these effects, as evidenced by the gradual upward trend in validation accuracy across epochs.

For EfficientNetV2-B0 (training curves in the code repository), the training accuracy rises above the validation accuracy across the five folds, while the training loss continues to decrease and the validation loss plateaus—a degree of overfitting expected for a dataset of this size. Restoration of the best-validation weights through early stopping selects the best-generalizing checkpoint for each fold. Because both architectures were trained under the same end-to-end protocol, neither model benefits from a training-strategy-specific convergence advantage; the observed differences reflect the backbones themselves.

Taken together, both models, trained under the same end-to-end protocol, exhibit a train–validation gap consistent with the limited dataset size (training curves in the code repository). MobileViT-XXS generalizes more stably across folds—reflected in its lower fold-to-fold variability—despite its substantially smaller parameter count. These observations are consistent with the cross-validation and test results presented in the subsequent sections, and are further discussed in Section 4.4.

### 3.2. Per-Fold and Class-Wise Performance

As indicated in Table 3, the MobileViT-XXS model showed higher accuracy in four of the five folds and lower performance variance than EfficientNetV2-B0; in fold 5, the two models were tied (0.6778). MobileViT-XXS achieved fold-wise accuracy in the range of 0.678–0.767, whereas EfficientNetV2-B0 exhibited greater variability, ranging from 0.522 to 0.711. In terms of ROC–AUC, MobileViT-XXS exceeded 0.90 in every fold and was the higher of the two models in four of the five folds (fold 5: 0.9076 vs. 0.9100). To further elucidate the class-level performance of the models, precision analysis was carried out at the grade level.

To characterize performance beyond overall accuracy—particularly given the clinical importance of early-stage detection—additional metrics were computed on the common test set and reported as the mean ± standard deviation (with 95% confidence interval) across the five folds. These across-fold dispersions (SD and 95% CI) quantify variability arising from model training across the five folds, all evaluated on the same test images, rather than sampling uncertainty over an independent patient population; the latter would require bootstrap resampling of test cases or external cohorts and is left for future work. EfficientNetV2-B0 achieved a balanced accuracy of 0.627 ± 0.079 (95% CI 0.529–0.726), a macro-averaged F1 of 0.617 ± 0.079, and an ordinal quadratic-weighted kappa (QWK) of 0.812 ± 0.069; the corresponding values for MobileViT-XXS were 0.707 ± 0.033, 0.703 ± 0.035, and 0.846 ± 0.030. Per-class sensitivity confirmed Grade 1 as the most challenging class, and here MobileViT-XXS was the more sensitive model (0.537 ± 0.180 versus 0.358 ± 0.164 for EfficientNetV2-B0), whereas Grade 0 and Grade 3 sensitivities were higher for both models (Grade 0 0.81–0.83; Grade 3 0.66–0.73) and per-class specificity remained ≥ 0.73 for all grades. The high QWK relative to overall accuracy reflects that misclassifications overwhelmingly involved adjacent grades: for the 5-fold ensemble, 87.5% of EfficientNetV2-B0 and 85.7% of MobileViT-XXS errors were to a neighboring grade, with only 12.5% and 14.3%, respectively, spanning two or more grades. Clinically, this predominance of adjacent-grade confusion—most frequently involving Grade 1 (with Grade 0 or Grade 2)—is consistent with the well-documented inter-observer variability of early hypertensive retinopathy grading. However, not all adjacent errors are clinically equivalent: Grade 1-versus-Grade 0 confusion (disease versus normal) can carry screening consequences and is examined separately through the binary referable-HR analysis below, whereas the rare non-adjacent (two-or-more-grade) errors represent larger grading discrepancies.

Table 4 shows the precision values for the EfficientNetV2-B0 and MobileViT-XXS models for the hypertensive retinopathy grades. The results show that MobileViT-XXS achieved higher precision than EfficientNetV2-B0 on all four grades (Grade 0 0.785 vs. 0.739; Grade 1 0.606 vs. 0.476; Grade 2 0.686 vs. 0.596; Grade 3 0.854 vs. 0.717), with the largest margins on the two hardest classes, Grade 1 and Grade 2.

Recall and F1-scores were also calculated for the respective grades. The detailed analysis of the performance of the models with regard to sensitivity will be discussed in the subsequent sections.

Table 5 reports the class-wise recall (sensitivity) for both architectures. MobileViT-XXS achieved higher sensitivity than EfficientNetV2-B0 on the three pathological grades—Grade 1 (0.537 vs. 0.358), Grade 2 (0.750 vs. 0.660), and Grade 3 (0.727 vs. 0.664)—while the two models were comparable on Grade 0 (0.814 vs. 0.828). The largest difference is on Grade 1, the clinically hardest class, where MobileViT-XXS retained markedly higher sensitivity despite its smaller capacity. Grade 1 nonetheless remained the weakest class for both models, reflecting the difficulty of distinguishing early hypertensive retinopathy from normal retinae.

Across the pathological grades, MobileViT-XXS provided the more sensitive operating profile; together with its substantially smaller footprint, this supports its selection for the edge-deployment setting.

### 3.3. Fold-Averaged and Ensemble Performance

Because a single evaluation strategy cannot fully characterize a model, two complementary approaches are reported in parallel: (i) fold-averaged (K-averaged) evaluation and (ii) 5-fold ensemble evaluation. The five-fold models were not evaluated on separate test partitions: each fold model was trained on four folds—with its held-out fold used only for validation monitoring—and was then evaluated on the same fixed 90-image, patient-disjoint internal test set. Accordingly, the fold-averaged (K-averaged) metrics report the mean ± standard deviation of the five models on this common test set, quantifying model-to-model variability, while the ensemble corresponds to averaging the five models’ softmax probability outputs (soft voting) into a single prediction per image.

The two summaries answer different questions. The fold-averaged summary describes how stable the training procedure is across data partitions, since it reports the spread of five independently trained models on one fixed test set. The ensemble summary describes the behavior of the system that is actually deployed, since the five models are combined by soft voting into a single prediction per image. They are computed from the same predictions on the same images and are therefore reported together for transparency rather than as independent evidence.

Fold-averaged metrics were obtained as the arithmetic mean and standard deviation of the five per-fold values on the common test set, with K = 5 folds.

Table 6 reports the resulting fold-averaged values alongside the ensemble values. MobileViT-XXS showed smaller standard deviations than EfficientNetV2-B0 on every reported metric, most clearly for ROC–AUC (0.009 vs. 0.026) and Cohen’s kappa (0.046 vs. 0.103), indicating more consistent behavior across data partitions. As stated in Section 2.2, these dispersions quantify variability introduced by the training partition and by stochastic optimization, not sampling uncertainty in diagnostic performance.

**Figure 5:**
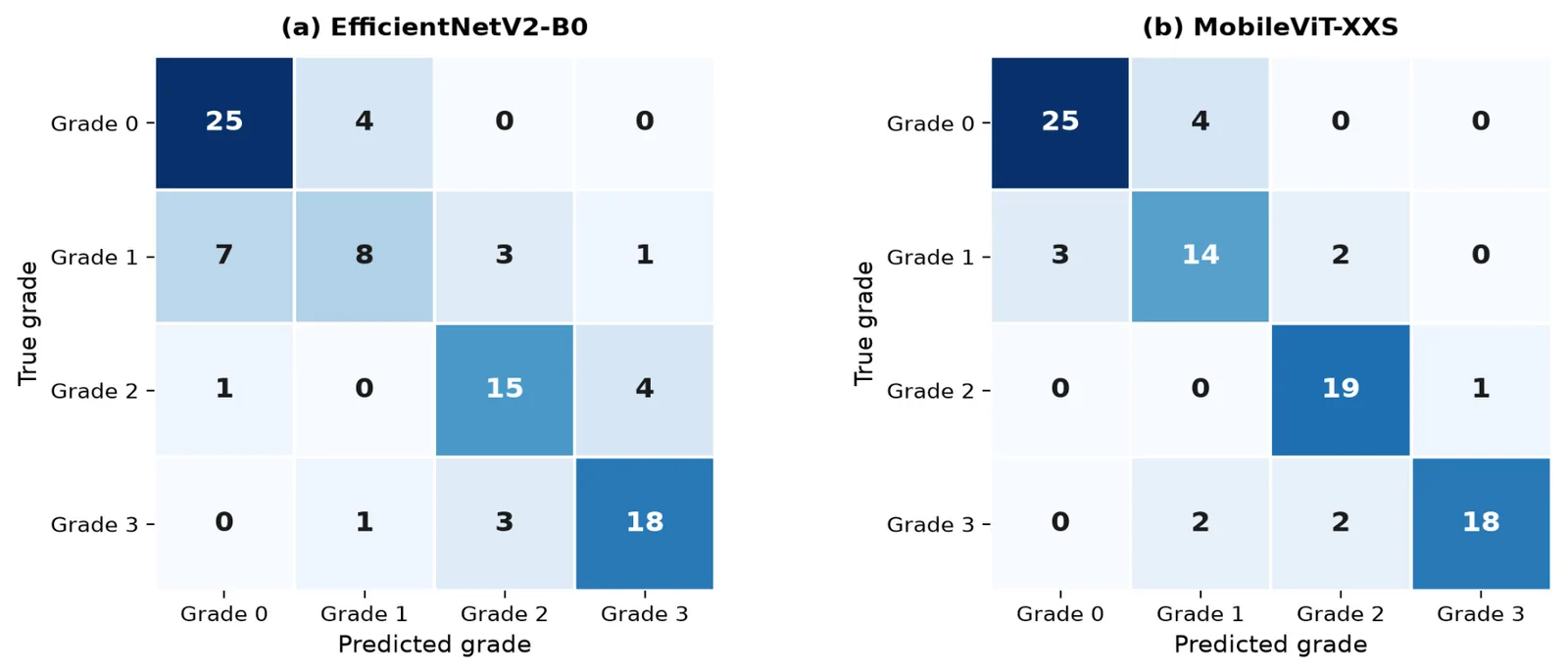
Five-fold soft-voting ensemble confusion matrices on the independent 90-image test set. (**a**) EfficientNetV2-B0; (**b**) MobileViT-XXS. Rows are true grades (Grade 0–3); columns are predicted grades. The full per-fold confusion matrices are provided in the code repository.

Table 6 reports both fold-averaged and 5-fold ensemble performance for the two models on the independent test set. For the fold-averaged evaluation, metrics were averaged across the five folds with standard deviations reported to capture fold-to-fold variability. For the ensemble evaluation, the per-class softmax outputs of the five fold models were averaged by soft voting to produce a single prediction per test image, reflecting the aggregate behavior of the deployed system. Figure 5 shows the resulting ensemble confusion matrices for both architectures on the 90-image, patient-disjoint test set. Both are strongly diagonal, and in both the Grade 1 row is the least concentrated: MobileViT-XXS assigns 14 of 19 Grade 1 images correctly against 8 of 19 for EfficientNetV2-B0, and the dominant Grade 1 error of EfficientNetV2-B0 is assignment to Grade 0 (7 of 19). Only 2 of 90 MobileViT-XXS predictions and 3 of 90 EfficientNetV2-B0 predictions fall more than one grade from the reference.

### 3.4. Ensemble Discrimination Performance (ROC Analysis)

To assess robustness against data partitioning, stratified five-fold cross-validation was conducted for both MobileViT-XXS and EfficientNetV2-B0. For this purpose, a 5-fold cross-validation was conducted on the available training dataset comprising 388 patient-level images. In the framework of this study, the dataset was divided into five folds, where four folds were used for the training set, and the remaining fold was used for the validation set. The process was repeated five times to ensure all instances were used for validation exactly once.

**Figure 6:**
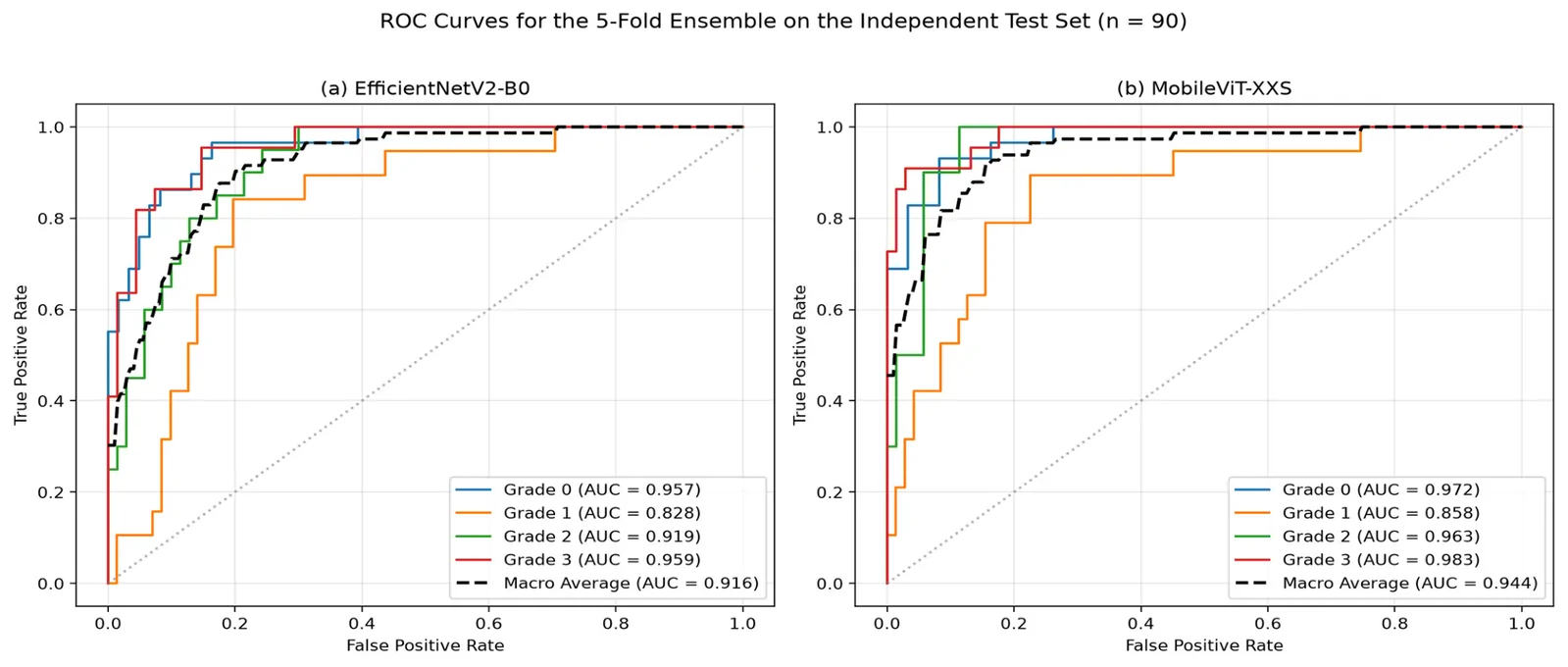
ROC curves for the 5-fold ensemble models on the independent test set (*n* = 90). (**a**) EfficientNetV2-B0; (**b**) MobileViT-XXS. Curves are shown for each hypertensive retinopathy grade (Grade 0–3) using a one-vs-rest strategy, along with the macro-average ROC curve.

The ROC curves for the 5-fold ensemble models on the independent test set are presented in Figure 6. These curves were computed using a two-step ensemble inference procedure. In the first step, each of the five trained fold models was applied independently to the full independent test set (*n* = 90), producing five separate per-class softmax probability matrices of shape (90, 4). In the second step, these five probability matrices were averaged element-wise to yield a single ensemble probability matrix, which was then used to compute the ROC curves and AUC values via the one-vs-rest (OvR) strategy. This approach mirrors the inference procedure deployed on the edge device (Section 2.7) and therefore reflects the discriminative performance of the system as it operates in practice. The resulting ensemble macro-average AUC values (MobileViT-XXS: 0.944; EfficientNetV2-B0: 0.916) are higher than the fold-averaged AUC values reported in Table 6 (0.916 and 0.880, respectively), which is expected: averaging probability outputs across multiple models reduces prediction variance and improves calibration. Both models demonstrate strong discriminative performance across all grades, with Grade 3 achieving the highest AUC values (MobileViT-XXS: 0.983; EfficientNetV2-B0: 0.959), while Grade 1 consistently yielded the lowest AUC for both architectures (0.858 and 0.828, respectively), consistent with the established clinical difficulty of mild-stage discrimination.

### 3.5. Comparative Performance and Statistical Analysis

This segment provides a comparative evaluation of the MobileViT-XXS and EfficientNetV2-B0 architectures using the average results from the 5-fold cross-validation and independent test set results. The evaluation covers not only accuracy but also generalization and robustness.

From the test-set analysis, it is apparent that the MobileViT-XXS model outperforms EfficientNetV2-B0 in terms of instance-level metrics such as accuracy, weighted F1-score, and Cohen’s kappa, indicating strong classification performance on the patient-disjoint test set. As a 5-fold ensemble, MobileViT-XXS reached 84.4% accuracy and a quadratic-weighted kappa of 0.916, reflecting strong ordinal agreement, with 97.8% of predictions falling within one grade of the reference label.

To assess whether these differences were statistically significant, the two architectures were compared across the five folds using a paired *t*-test (Section 2.6). MobileViT-XXS was numerically superior to EfficientNetV2-B0 on every metric and in nearly all folds (accuracy 0.720 vs. 0.651; weighted F1 0.716 vs. 0.637; Cohen’s kappa 0.623 vs. 0.527); with only five folds the paired difference approached but did not reach significance (*p* = 0.06–0.08), while the Wilcoxon signed-rank test showed the same consistent direction, MobileViT-XXS winning all five folds on weighted F1 and Cohen’s kappa. On the independent test set, a sample-level McNemar’s test confirmed that MobileViT-XXS significantly outperformed EfficientNetV2-B0 (*p* = 0.013). The two models achieved comparably high ordinal grading consistency (quadratic-weighted kappa), while MobileViT-XXS retained the instance-level advantage.

### 3.6. On-Device Inference Performance

Table 7 presents the 5-fold ensemble inference performance of both models on the NVIDIA Jetson Orin Nano Developer Kit at FP32 precision, measured through the Gradio-based web interface. Two conditions were recorded for each model: a cold start, representing the first inference after device initialization where all five fold models are loaded simultaneously alongside GPU warm-up, and a warm start, representing subsequent stable inference with the models already resident in memory. The cold start times of 6275.7 ms (0.16 FPS) for MobileViT-XXS and 4128.4 ms (0.24 FPS) for EfficientNetV2-B0 reflect the one-time GPU initialization overhead inherent to TensorFlow on the Jetson platform. Following warm-up, ensemble inference stabilized at 485.8 ms (2.06 FPS) for MobileViT-XXS and 420.4 ms (2.38 FPS) for EfficientNetV2-B0, demonstrating comparable interactive-speed ensemble inference for both architectures on compact edge hardware. Beyond the FP32 ensemble benchmarks, TensorRT FP16 optimization was applied to single-fold models of both architectures on the same device using an ONNX-based conversion pipeline (SavedModel → ONNX opset 13 → TensorRT FP16 engine). Single-model TensorRT FP16 inference achieved 3.56 ± 0.91 ms (280.84 FPS) for EfficientNetV2-B0 and 3.76 ± 0.90 ms (265.67 FPS) for MobileViT-XXS, representing 22.7× and 27.2× speedups over their respective FP32 single-model baselines. The deployed five-fold soft-voting ensemble, profiled over 1500 consecutive inferences, sustained 11.35 ± 1.09 ms (88 FPS) for EfficientNetV2-B0 and 10.40 ± 1.14 ms (96 FPS) for MobileViT-XXS—well within real-time screening limits.

To further characterize deployment feasibility, resource utilization was profiled during sustained five-fold ensemble TensorRT FP16 inference (1500 consecutive ensemble inferences per architecture) on the Jetson Orin Nano operated in the 25 W (MAXN_SUPER) power mode. Sustained steady-state ensemble latency was 11.35 ms (88 FPS, p95 13.1 ms) for EfficientNetV2-B0 and 10.40 ms (96 FPS, p95 13.1 ms) for MobileViT-XXS over 1500 consecutive ensemble inferences; latency remained stable across the run, with the final fifth of inferences no slower than the first, indicating no thermal throttling. Peak unified-memory usage was 3.9–4.0 GB of the 7.6 GB available; mean CPU utilization remained below 14%, mean GPU utilization was ≈72%, and board-level power draw (VDD_IN) averaged 8.85–8.92 W (peak 9.8–10.2 W). No thermal throttling was observed—latency decreased rather than increased over the 1500-inference run—confirming that both models operate well within the compute, memory, and power envelope of the device and sustain real-time throughput during continuous operation.

### 3.7. Comparison with Published Studies

N/R: not reported; DSC: depth-wise separable convolution; SCM: spatial convolution module; HHO: Harris Hawk optimization; HR: hypertensive retinopathy; DR: diabetic retinopathy; KWB: Keith–Wagener–Barker; OCT: optical coherence tomography. Accuracy values reflect the best reported result per study. Direct performance comparisons across studies are limited due to differences in imaging modality, task definition, dataset size, and validation strategy.

## 4. Discussion

This study compares MobileViT-XXS and EfficientNetV2-B0 for automated multi-class grading of hypertensive retinopathy. MobileViT-XXS is a lightweight CNN–Transformer hybrid architecture that integrates local convolutional features with global contextual modeling. The main objective of this research paper is not only to achieve high accuracy but also to create a balanced decision-making process.

### 4.1. Comparison of Overall Performance with Literature

The results indicate strong discriminative performance for hypertensive retinopathy grading. Under rigorous patient-level 5-fold cross-validation, MobileViT-XXS achieved 72.0% fold-averaged accuracy and a fold-averaged macro ROC–AUC of 0.916, rising to 84.4% accuracy and 0.944 macro ROC–AUC as a five-fold soft-voting ensemble; the corresponding values for EfficientNetV2-B0 were 65.1% and 0.880 fold-averaged, and 73.3% and 0.916 as an ensemble. Table 8 places these results in context: alongside Şüyun et al. [12], the present study is the only one using OCT rather than fundus photography, and the only one addressing the four-grade KWB-inspired task on en-face OCT exclusively. Şüyun et al. [12] reported a higher accuracy (94.66%) on an image-level version of the same imaging data, but used a three-stage pipeline (14 CNN architectures, feature fusion, machine-learning classifiers, and Harris Hawk optimization) without cross-validation—a computationally intensive design inherently unsuitable for edge deployment. The lower accuracy of the present models is therefore not a regression but an expected consequence of prioritizing lightweight, edge-deployable architectures; the two studies are complementary, addressing the same clinical problem at opposite ends of the accuracy–deployability spectrum. Studies reporting higher accuracy do so under different conditions. Abbas et al. report 98% on a three-class normal/HR/diabetic retinopathy task [7]. Bhimavarapu et al. report 98.99% on a five-class fundus-based hypertensive retinopathy task with data augmentation [11]. Suman et al. report 96.88% on a four-grade fundus task evaluated with an image-level single split and no patient-level grouping [15]. None reports patient-disjoint cross-validation or on-device inference, making direct comparison with the present four-grade OCT task methodologically inappropriate. The macro ROC–AUC values (0.88–0.94) confirm strong multi-class discrimination; because ROC–AUC is threshold-independent, the operating point can be tuned toward higher sensitivity or specificity per the clinical setting. These results are consistent with prior CNN-based retinal work [8]. They are equally consistent with lightweight transformer frameworks for retinal disease classification from OCT [28]. Hybrid CNN–Transformer designs for retinal imaging report the same balance of accuracy against computational cost [29]. Comparable end-to-end pipelines have been reported outside ophthalmology, for instance for pulpal calcification detection on bite-wing radiographs [30]. What the present work adds is validated near-real-time inference on a compact edge device, which, to the authors’ knowledge, none of the compared studies report.

### 4.2. Interpretation of Fold-Based and Ensemble Results

In five-fold cross-validation, MobileViT-XXS showed lower performance variability across folds—with smaller standard deviations in accuracy (0.035 vs. 0.075), Cohen’s kappa, and ROC–AUC than EfficientNetV2-B0 (Table 6)—and also generalized better on the independent test set despite its substantially smaller parameter count. Its local–global feature coupling appears to support stable, consistent learning under patient-level stratified splitting. Fold-averaged and ensemble evaluations are complementary: the former captures generalization stability across partitions, the latter the aggregate discriminative power of the deployed system.

The advantage of the five-model ensemble over its constituent fold models is consistent with reports that ensembling convolutional backbones improves diagnostic accuracy in retinal imaging [31]. Ensembles that combine convolutional and vision-transformer members have been reported to help for the same reason in other medical-imaging tasks, where the two families make partly complementary errors [32].

### 4.3. Class-Based Performance and Clinical Significance

When the outcomes are examined by discrete grade level, the performance of the two models was excellent for Grade 0 (Normal) and Grade 3 (Severe HR). The models more easily identified the unique vascular abnormalities, hemorrhage, and fluid accumulation in the late-stage HR cases.

Distinguishing Grade 1 from Grade 2 was more challenging, and the two were frequently confused—mirroring the clinical setting, where early and moderate HR can appear similar (mild vessel narrowing and arteriovenous crossing changes) [5]. The confusion matrices (Figure 5; per-fold detail in the code repository) quantify this: Grade 1 recall was 0.358 for EfficientNetV2-B0 and 0.537 for MobileViT-XXS (Table 5). Two failure modes emerged. The first is Grade 1–Grade 2 confusion, driven by the genuine visual similarity between mild arteriolar narrowing and early arteriovenous nicking—findings also subject to high inter-observer disagreement in clinical fundoscopy [17]. The second, more pronounced mode is Grade 1–Grade 0 confusion, where subtle early-stage vascular changes fail to produce discriminative activations; this was more marked for EfficientNetV2-B0, whose lower Grade 1 sensitivity reflects greater difficulty separating early changes from normal retinae, consistent with the broader literature on early-stage HR detection [19].

Because four-class accuracy is constrained by the intrinsic overlap of the early grades, we additionally evaluated the two clinically decisive binary decisions on the 5-fold ensemble, with 95% confidence intervals obtained by bootstrap resampling of the 90-image test set. For referable-HR triage (Grade ≥ 2), MobileViT-XXS reached 95.6% accuracy (95% CI 91.1–98.9), 95.2% sensitivity (87.8–100), 95.8% specificity (89.6–100), and a 95.8% negative predictive value (89.4–100), with an area under the curve of 0.986 (0.957–1.000); EfficientNetV2-B0 was comparable (accuracy 93.3%, sensitivity 95.2%, NPV 95.7%, AUC 0.986). For severe-HR triage (Grade 3), MobileViT-XXS achieved 98.5% specificity (95.4–100) and 94.4% NPV at 81.8% sensitivity (AUC 0.983), and EfficientNetV2-B0 92.7% specificity and 94.0% NPV (AUC 0.959). The high referable-HR sensitivity (95.2%), reflected in a correspondingly high NPV, indicates that few patients with referable disease would be missed at the operating threshold, supporting use of the system as a triage aid. Consistent with the ensemble’s variance reduction, its per-class Grade 1 sensitivity (MobileViT-XXS 0.737, 95% CI 0.533–0.917) exceeded the fold-averaged value. Although both models were strongly discriminative for referable-HR (AUC ≈ 0.99), their probability outputs were only moderately calibrated (expected calibration error 0.209 for MobileViT-XXS and 0.224 for EfficientNetV2-B0; Brier scores 0.085 and 0.093), tending to underestimate the referable-HR probability in the mid-range (reliability curves in the code repository). Triage therefore relies on the thresholded class decision rather than the raw probability, and post-hoc calibration (e.g., temperature scaling) is recommended before any probability-based clinical use. These estimates derive from a single-center test set of 90 images and require external, multi-center confirmation.

### 4.4. Training Dynamics and Overfitting Analysis

Both models—trained under the same end-to-end protocol with early stopping and best-validation-weight restoration—show a train–validation gap consistent with the limited dataset size (Section 3.1). Across folds, MobileViT-XXS exhibits lower variability and relatively consistent validation accuracy (approximately 0.50–0.62), indicating more stable generalization despite its smaller parameter count; the augmentation strategy and focal loss with balanced class weights further support learning of the minority grades. This fold-level consistency is valuable in clinical contexts where reproducible output matters even when overall accuracy is limited.

### 4.5. Edge Device Applicability and Practical Contribution

The inference benchmarks (Section 2.7) confirm stable warm-start latency on the Jetson Orin Nano, with TensorRT FP16 five-fold ensemble inference of ≈10–11 ms and single-model inference under 4 ms for both architectures—a 22–27× speedup over their FP32 baselines. The key deployment finding is the trade-off profile: MobileViT-XXS delivers comparable ensemble latency to EfficientNetV2-B0 at less than one-fifth of the model size (3.83 vs. 23.70 MB) and roughly one-sixth of the parameter count, which is particularly relevant where storage, memory bandwidth, and update overhead are limiting. These results show that lightweight CNN–Transformer hybrids are viable alternatives to larger convolutional models for on-device inference, without cloud connectivity or high-end GPU infrastructure. Given the low Grade 1 sensitivity, the models are best positioned as a decision-support and triage aid—particularly for flagging referable (Grade ≥ 2) and severe (Grade 3) disease—rather than as a standalone screening tool. Because the pipeline is architecture-agnostic and data-driven, it can transfer to other medical-imaging tasks across modalities [33].

Beyond this technical feasibility, the intended clinical value of an edge-deployable HR grading tool lies in widening access to retinal assessment. A compact, offline-capable system could support opportunistic screening and triage in primary-care and community settings, help flag severe (Grade 3) cases for prompt ophthalmological referral, and extend teleophthalmology workflows to resource-limited or remote centers where specialist coverage and reliable connectivity are scarce. Used as a decision-support aid rather than a standalone diagnosis—and with its early-stage (Grade 1) limitations clearly communicated—such a tool could help prioritize which patients require specialist review.

The same case has been made for automated diabetic retinopathy screening in low- and middle-income settings, where the binding constraint is access rather than algorithmic accuracy [34]. Interface design is not incidental to that role: osteoporosis staging on panoramic radiographs has been delivered together with a purpose-built graphical user interface for precisely this reason [35]. Localization of all impacted teeth with prediction of third-molar Winter angulation follows the same model-plus-interface pattern [36]. Server-side delivery of a lightweight attention-based classifier to a thin client has likewise been demonstrated as a practical deployment route [37].

### 4.6. Limitations and Future Work

There are some limitations to this study. First, the data originate from a single institution (Batıgöz Hospital, İzmir) under ethics approval (No. 2025/281); although the dataset spans the full range of KWB-inspired grades (Grade 0–3) encountered in routine screening, generalization to other OCT devices, camera systems, and populations remains to be confirmed in external, multi-center studies. All partitions—the training/test division and the five cross-validation folds—were performed at the patient (subject) level, so that no patient contributed images to both training and test and subject-level leakage was eliminated; however, the held-out test set is internal to the same institution and acquisition protocol and therefore does not constitute external validation. Second, discriminating the early stages (Grade 1 vs. Grade 2, and Grade 1 vs. Grade 0) remained the principal difficulty, consistent with the known clinical ambiguity of early hypertensive retinopathy; vessel-segmentation or lesion-based explainability may help clarify these boundaries. Third, the interpretability analysis is qualitative and is subject to three specific constraints. (a) Grad-CAM for MobileViT-XXS was computed on the final convolutional feature map of the network (the output projection after the last MobileViT block), since Grad-CAM cannot be applied to the folded patch-token tensors inside the MobileViT blocks; a single gradient-weighted convolutional map therefore summarizes the class-discriminative response at a coarse spatial resolution and does not directly depict the token-level self-attention interactions themselves. Transformer-compatible methods (e.g., attention rollout, Grad-CAM++) are left for future work. (b) The maps were generated from the fold-1 model of each architecture, whereas every performance figure reported here is that of the five-fold soft-voting ensemble; single-model attribution is not a substitute for ensemble-level attribution, which remains an open methodological item. (c) Because the dataset carries image-level grades only and no pixel-wise lesion annotation, attention–pathology agreement could be assessed only qualitatively (Section 4.7) and no localization metric could be computed; a dedicated lesion-annotation campaign, with the resulting masks used both to quantify overlap and to compare attribution methods, is the necessary next step, and is the principal reason the present system is positioned for ordinal grading and referable-HR triage rather than for lesion localization. Fourth, the primary latency benchmarks were obtained at FP32; TensorRT FP16 optimization reduced single-model inference to 3.56 ms (EfficientNetV2-B0) and 3.76 ms (MobileViT-XXS), and the five-fold ensemble to 11.35 ms and 10.40 ms, respectively, without measurable accuracy loss, but INT8 quantization for this fine-grained four-grade task remains to be evaluated. Fifth, moving from ordinal grading toward lesion-level, multi-label detection of individual HR findings (e.g., cotton-wool spots, hard exudates, arteriovenous nicking, retinal hemorrhages) would yield more directly interpretable output but requires lesion-level annotations. Sixth, the analysis used en-face OCT alone; incorporating complementary B-scan and fundus views together with systemic data (e.g., blood pressure and glycemic status) may improve grading confidence, particularly where diabetic retinopathy findings overlap. Finally, the five-fold models were combined by uniform soft voting; weighted voting schemes whose member weights are optimized rather than fixed have been reported to improve ensemble performance in multi-disease retinal classification [38], and represent a direct extension of the present aggregation step.

### 4.7. Grad-CAM Analysis: Attention–Pathology Concordance and Failure Cases

**Figure 7:**
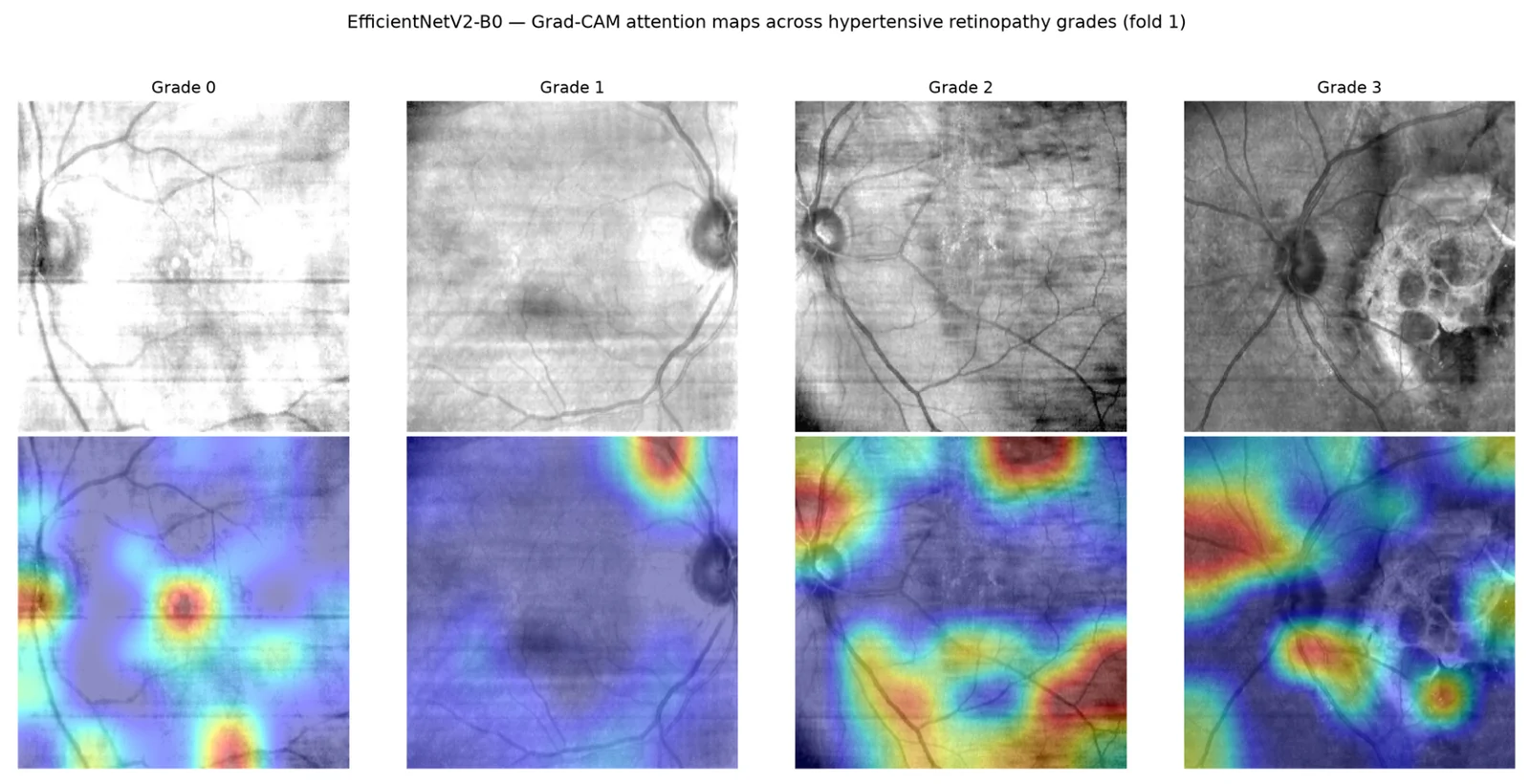
EfficientNetV2-B0 Grad-CAM attention maps across hypertensive retinopathy grades, computed from the fold-1 model on the final Conv2D layer. Top row: source en-face OCT images; bottom row: the corresponding Grad-CAM overlays (jet color map, red denoting the highest normalized activation within each map). Columns show one representative case per grade (Grade 0–3); the same four source images are used in Figure 8. Warm regions indicate where the network responds most strongly and are not a validated lesion location: no pixel-level reference annotation exists for this dataset, and both concordant and discordant cases are analyzed in Section 4.7 and Table 9.

**Figure 8:**
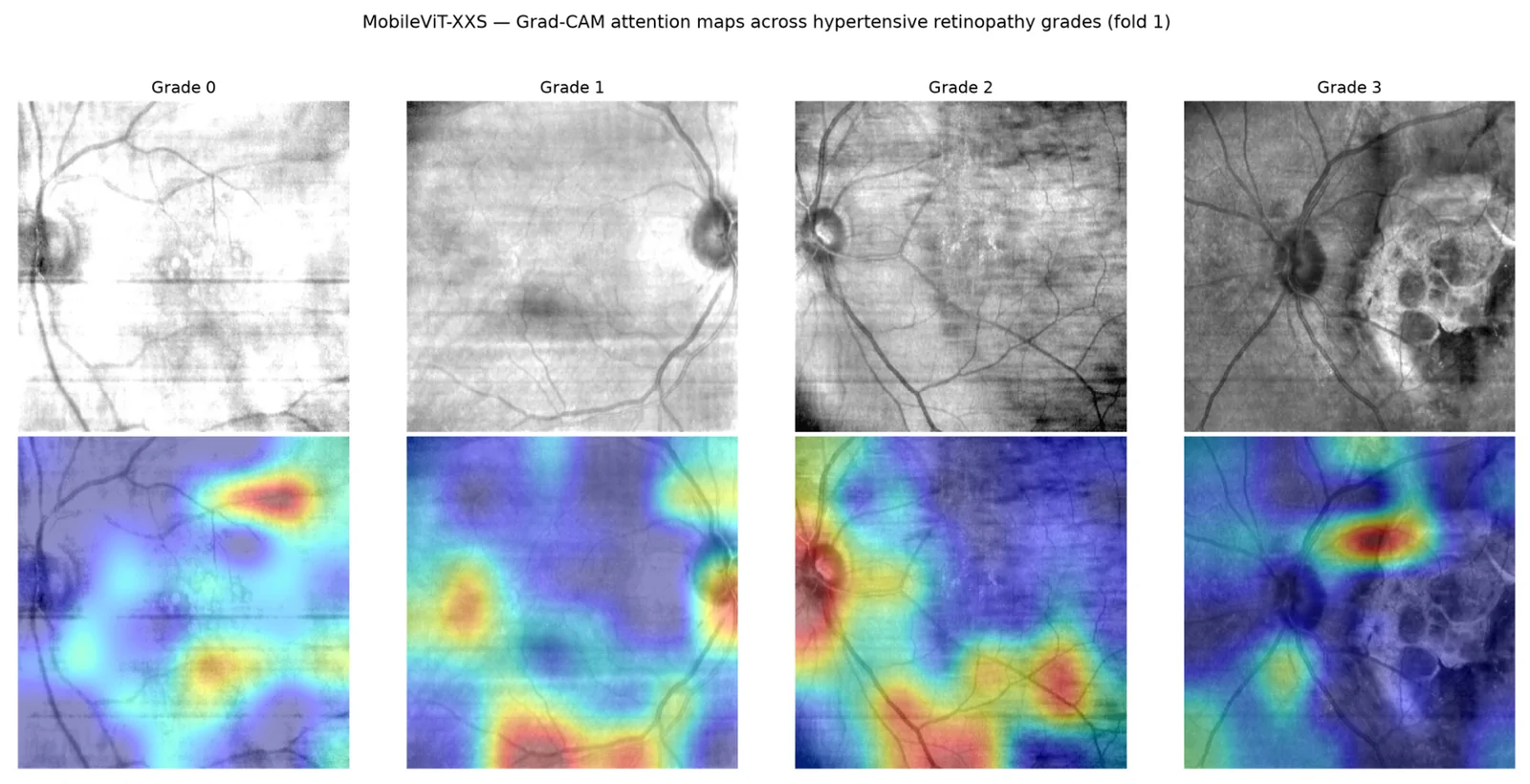
MobileViT-XXS Grad-CAM attention maps across hypertensive retinopathy grades, computed from the fold-1 model on the final convolutional feature map (after the last MobileViT block). Top row: source en-face OCT images (identical to Figure 7); bottom row: the corresponding Grad-CAM overlays (jet color map, red denoting the highest normalized activation within each map). Because a single gradient-weighted convolutional map cannot depict the token-level self-attention that also contributes to the final prediction, these maps are a coarse class-discriminative summary and are not a validated lesion location (Section 2.8 and Section 4.7 and Table 9).

Grad-CAM visualizations were produced for both models from their fold-1 weights (EfficientNetV2-B0: Figure 7; MobileViT-XXS: Figure 8; summarized in Table 9). The same four source images were used for both architectures, so that the two models can be compared directly on identical inputs. For EfficientNetV2-B0, Grad-CAM on the last Conv2D layer produced spatially coherent maps whose activation is most extensive for the two higher grades, broadly consistent with the vascular severity progression of the KWB-inspired scale, although the trend is not strictly monotonic across the four illustrated cases: the Grade 1 example elicits a more restricted response than the Grade 0 example. For MobileViT-XXS, Grad-CAM was computed on the final convolutional feature map of the network (after the last MobileViT block); the resulting maps are more broadly distributed. They too show more extensive activation at the higher grades, particularly for Grade 3, but because a single gradient-weighted convolutional map summarizes only the final class-discriminative response and cannot depict the token-level self-attention that also shapes the decision, they should be interpreted with caution rather than as a direct explanation of the final prediction.

Two distinctions are required before these maps can be read as evidence of clinical utility. The first is between classification success and localization success: a model may assign the correct grade while responding to regions a clinician would not inspect, and it may respond to the appropriate region while assigning the wrong grade. The second is between qualitative and quantitative localization assessment. As stated in Section 2.8, the present dataset carries image-level grades only, so no lesion reference exists against which overlap could be measured. The analysis below therefore reports attention–pathology concordance—the qualitative spatial agreement between the attention map and the structural findings visible on the corresponding source image—and presents both concordant and discordant examples rather than only favorable ones.

Concordant behavior is clearest in the Grade 2 example, where the strongest responses of both architectures follow the course of the major retinal vessels. EfficientNetV2-B0 (Figure 7, third column) responds along the inferior vascular arcade and across the vessel-dense region below the horizontal midline, and MobileViT-XXS (Figure 8, third column) responds on the optic disc and its emerging vessels in addition to the inferior arcade. This is the pattern expected of a model that has learned criteria defined on the caliber, course, and crossing behavior of the retinal arterioles rather than on any discrete lesion. The Grade 1 example shows a weaker but comparable pattern for EfficientNetV2-B0, whose single dominant response lies immediately superior to the optic disc (Figure 7, second column)—within the peripapillary zone in which retinal arteriolar caliber is conventionally quantified—while the remainder of the field is suppressed.

Discordant behavior is evident in three situations. First, and most importantly, in the Grade 3 example the most conspicuous structural abnormality on the source image—the lobulated, cyst-like region occupying the right-hand portion of the field—receives comparatively low activation in both architectures, while the dominant response falls in the superior part of the field, away from that region (Figure 7 and Figure 8, fourth column). The severe grade is thus reached without the map pointing at the finding that most obviously distinguishes the image. Second, in the Grade 0 example, EfficientNetV2-B0 produces several discrete high-activation foci over structurally unremarkable background retina, including regions traversed by the horizontal banding artefact characteristic of volumetric en-face acquisition (Figure 7, first column); MobileViT-XXS shows a similarly non-specific distribution (Figure 8, first column). A normal image has no target region by definition, so any focal response there is spurious by construction; the concern is that the same non-specific mechanism may also contribute to responses on pathological images. Third, and more mildly, part of the EfficientNetV2-B0 response in the Grade 2 example falls on an area of locally reduced en-face reflectivity in the superior field rather than on vascular structure.

Five factors plausibly account for this combination of vessel-following concordance and lesion-level discordance. (i) Spatial granularity: as noted in Section 2.8, a single Grad-CAM cell at the final convolutional stage of EfficientNetV2-B0 spans roughly one-seventh of the field of view in each direction, which is one to two orders of magnitude coarser than a cotton-wool spot or a focal hard exudate, so discrete lesions cannot be resolved even in principle. (ii) Objective mismatch: both networks were optimized for ordinal grade discrimination under a categorical focal loss with no localization supervision whatsoever, and the features that best separate adjacent grades need not coincide with the findings a clinician names when justifying a grade; diffuse texture and vessel-caliber statistics distributed across the field can be more discriminative than any single lesion, and gradient-based attribution follows whichever signal the classifier actually uses. (iii) The structure of the grading criteria: Grades 1 and 2 are defined by generalized and focal arteriolar change distributed along the branching vasculature rather than by discrete foci, so broad, vessel-following activation is the appropriate response at these grades and the absence of a single “suspected area” partly reflects the disease definition rather than a model deficiency; this argument does not extend to Grade 3, where discrete hemorrhages and exudates are present and a localized response would legitimately be expected. (iv) Single-fold attribution: the maps derive from fold-1 models, and for EfficientNetV2-B0 fold 1 was the weakest of the five (accuracy 0.522 against an ensemble accuracy of 0.733; Table 3 and Table 6), so its maps are not representative of the system whose performance is reported; the MobileViT-XXS fold-1 model (0.700) likewise sits below both its fold average (0.720) and the ensemble (0.844). (v) Method–architecture mismatch for MobileViT-XXS: although the map is taken at the final convolutional feature map, downstream of the transformer blocks, a single gradient-weighted convolutional map reduces the decision to one coarse spatial response and cannot depict the token-level self-attention interactions that MobileViT uses to combine information across the field, so its broad appearance is expected and is not evidence that the full model attends broadly.

To assess clinical plausibility, the Grad-CAM maps were qualitatively reviewed by a board-certified ophthalmologist (co-author), who reported that high-activation regions generally overlapped with the retinal vascular findings of hypertensive retinopathy and, in part, with regions clinicians attend to during grading (such as areas of edema or hemorrhage); discrete focal lesions—in particular exudative changes—were only partially captured and were not consistently localized by the attention maps. The MobileViT-XXS maps (Figure 8) corresponded well with vessel-dense regions; although broadly distributed, this breadth is consistent with the spatially distributed nature of HR vascular findings, which extend along the branching vasculature rather than concentrating at a single focus. Activation was most extensive for Grade 3, although it reflected broad vessel-dense regions rather than precise localization of individual lesions such as focal exudates. This expert appraisal is consistent with clinically relevant attention at the higher grades, but it is a qualitative visual assessment by a single observer rather than a quantitative lesion-localization validation, and it does not establish that either model localizes individual findings reliably.

Taken together, the maps support a deliberately limited claim: both networks respond preferentially to vascular and vessel-adjacent structure at the higher grades, which is consistent with—but is not proof of—clinically appropriate reasoning. They do not establish lesion-level localization, and the system must not be presented or used as a lesion-detection or lesion-localization tool. Its intended role remains ordinal grading and referable-HR triage (Section 4.3), for which the classification metrics reported in Section 3, and not the attention maps, are the relevant evidence. Class-activation mapping is widely used for this purpose in radiographic artificial intelligence, including Eigen-CAM explainability mapping for ordinal staging of periodontal bone loss on bite-wing radiographs [21]. It carries the same caveat in every setting: such maps indicate where a network responds, not whether its reasoning is clinically valid, and they cannot by themselves establish that the model has learned the diagnostic features a clinician would use.

**Figure 9:**
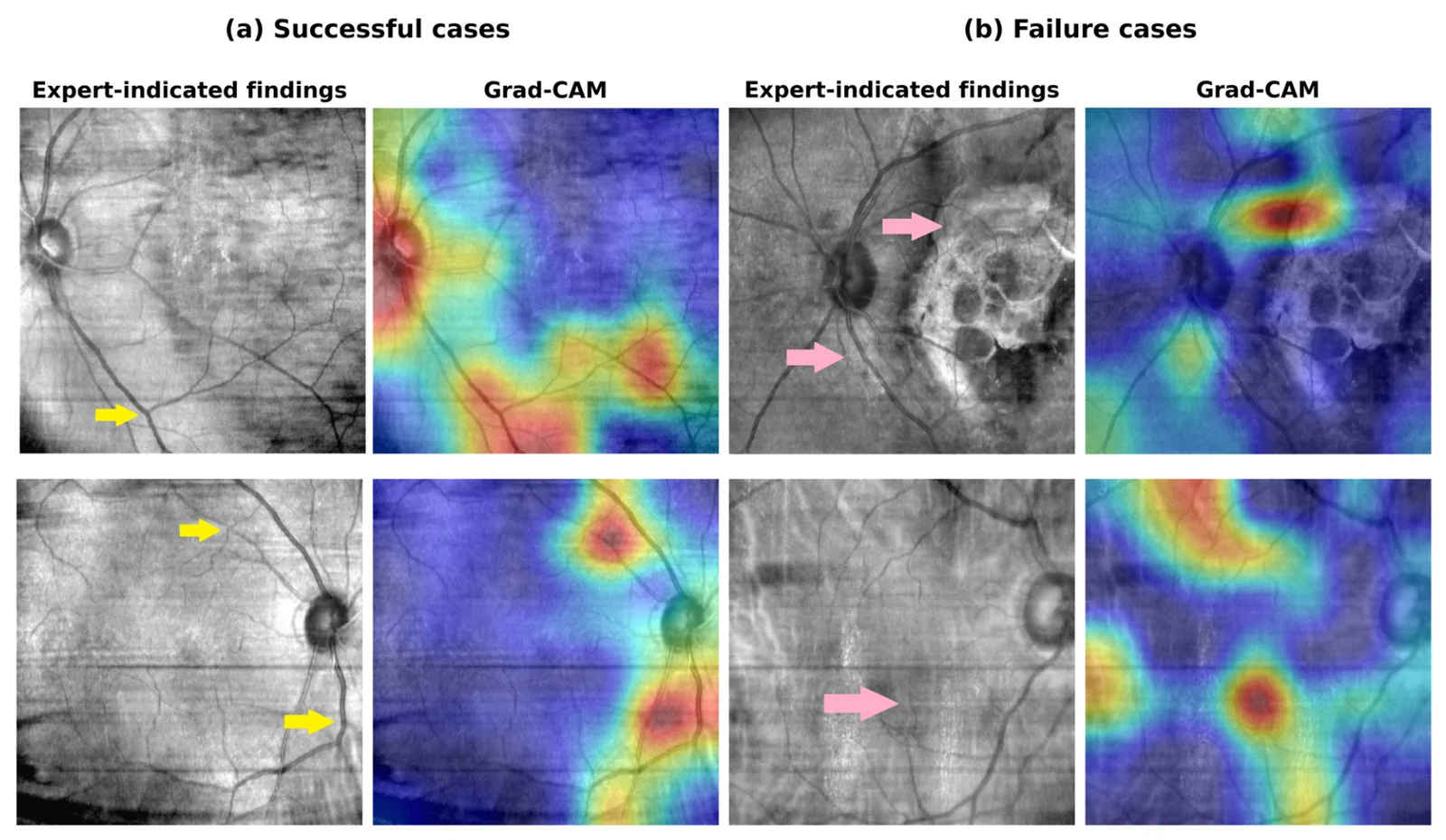
Case-level success–failure analysis of Grad-CAM attention (fold-1 models, computed with the procedure described in Section 2.8). Each pair presents the source en-face OCT image alongside the corresponding Grad-CAM overlay. Yellow arrows mark visible structural findings indicated by the ophthalmologist co-author; pink arrows mark regions that attract strong model attention although, on expert assessment, they do not contain strongly discriminative hypertensive retinopathy findings. (**a**) Successful cases: two correctly classified images in which the strongest activation follows the optic disc, the emerging vessels, and the arcades, concordant with the indicated findings. (**b**) Failure cases: a correctly classified image in which the dominant response falls on weakly discriminative regions while the conspicuous lobulated, cyst-like complex receives comparatively low activation, and an image whose severity is under-estimated with the attention again concentrated on weakly discriminative regions. All arrow annotations are qualitative expert indications, not pixel-level lesion annotations, and no localization metric is implied.

To make this success–failure contrast directly inspectable at the level of individual findings, Figure 9 pairs four representative cases with the same source images annotated by the ophthalmologist co-author: yellow arrows mark visible structural findings, whereas pink arrows mark regions that attract strong model attention although, on expert assessment, they do not contain strongly discriminative hypertensive retinopathy findings. In the two successful cases (Figure 9a), both correctly classified, the strongest activation follows the optic disc, the emerging vessels, and the vascular arcades—structures central to the clinical assessment of hypertensive retinopathy—and the maps are concordant with the indicated findings. In the two failure cases (Figure 9b), the maps make the cause of each failure visible: in the first, the classification is correct, yet the dominant response falls on weakly discriminative regions while the conspicuous lobulated, cyst-like complex receives comparatively low activation—the model reaches the right answer without attending to the most informative finding; in the second, the attention again concentrates on weakly discriminative regions and the severity of the image is under-estimated. Together the four cases state the central message of the section: the maps can be structurally plausible where the disease expresses itself as distributed vascular change, and can fail by directing attention to regions of little clinical informativeness—reaching a correct classification without attending to the most informative finding in one case and under-estimating severity in the other—reflecting the coarse attribution grid and the objective mismatch between classification and lesion localization.

## 5. Conclusions

This study presented a comparative evaluation of MobileViT-XXS and EfficientNetV2-B0 for four-grade hypertensive retinopathy classification from en-face OCT under stratified, patient-level 5-fold cross-validation with an independent test set. Under this leakage-free evaluation, MobileViT-XXS outperformed EfficientNetV2-B0 on the principal overall classification metrics (accuracy, weighted F1, quadratic-weighted kappa, and ROC–AUC) and showed lower fold-to-fold variability (Cohen’s kappa and ROC–AUC standard deviations two to three times smaller). Combined with its substantially smaller footprint, this makes MobileViT-XXS the more accurate, stable, and deployment-friendly choice for on-device HR triage and decision support.

Grade 1–Grade 2 discrimination remained the principal challenge for both architectures, consistent with the known inter-observer variability of early-stage HR grading. Error analysis identified two failure modes: Grade 1–Grade 2 confusion from genuine visual overlap, and the more prevalent Grade 1–Grade 0 confusion from the weak discriminative signal of subtle early changes. EfficientNetV2-B0 showed the lower Grade 1 sensitivity, more often misclassifying Grade 1 as Normal, whereas MobileViT-XXS retained higher Grade 1 sensitivity despite its smaller capacity. The accompanying Grad-CAM analysis (Section 4.7) showed attention concentrated on vascular and vessel-adjacent structures at the higher grades, together with clearly discordant cases in which the strongest response fell away from the most conspicuous abnormality; the maps are therefore reported as a qualitative plausibility check only, and the system should not be used to localize individual hypertensive retinopathy findings.

To the authors’ knowledge, this is among the first demonstrations of end-to-end four-grade HR grading from en-face OCT with 5-fold ensemble inference on a compact edge device, without cloud infrastructure or high-end GPU hardware. MobileViT-XXS offers a compelling size–performance trade-off, particularly where memory and bandwidth are limiting.

Collectively, these findings indicate that lightweight CNN–Transformer hybrids are promising, deployable alternatives to computationally intensive pipelines for HR grading in resource-limited settings, provided early-stage limitations are transparently communicated. The models are not yet clinically validated; external validation across devices, institutions, and populations is required before deployment. Future work should prioritize multi-center validation, transformer-compatible explainability (e.g., attention rollout), on-device INT8 optimization, and prospective evaluation of the Gradio interface. More broadly, the demonstrated pipeline is architecture-agnostic and transferable to other imaging tasks, providing a reproducible foundation for point-of-care edge AI.

## Figures and Tables

**Table 1:** Class-wise image distribution in the training set.

Class	Number of Images
Grade 0	96
Grade 1	106
Grade 2	95
Grade 3	91
Sum	388

**Table 2:** Class-wise image distribution in the test set.

Class	Number of Images
Grade 0	29
Grade 1	19
Grade 2	20
Grade 3	22
Sum	90

**Table 3:** Per-fold classification performance of EfficientNetV2-B0 and MobileViT-XXS on the independent 90-image test set (each fold model evaluated on the same test set; these are not cross-validation held-out results).

Fold	Model	Accuracy	F1-Score (Weighted)	Cohen’s Kappa	ROC–AUC (OvR)
Fold 1	EfficientNetV2-B0	0.5222	0.5052	0.3483	0.8376
Fold 1	MobileViT-XXS	0.7000	0.6965	0.5959	0.9273
Fold 2	EfficientNetV2-B0	0.6556	0.6489	0.5368	0.8782
Fold 2	MobileViT-XXS	0.7111	0.6910	0.6055	0.9057
Fold 3	EfficientNetV2-B0	0.6889	0.6567	0.5775	0.8883
Fold 3	MobileViT-XXS	0.7444	0.7393	0.6551	0.9187
Fold 4	EfficientNetV2-B0	0.7111	0.6981	0.6073	0.8859
Fold 4	MobileViT-XXS	0.7667	0.7652	0.6852	0.9210
Fold 5	EfficientNetV2-B0	0.6778	0.6779	0.5657	0.9100
Fold 5	MobileViT-XXS	0.6778	0.6863	0.5717	0.9076

**Table 4:** Class-wise precision values (five-fold mean ± SD).

Grade	EfficientNetV2-B0 Precision (Mean ± SD)	MobileViT-XXS Precision (Mean ± SD)
Grade 0	0.739 ± 0.060	0.785 ± 0.052
Grade 1	0.476 ± 0.122	0.606 ± 0.160
Grade 2	0.596 ± 0.101	0.686 ± 0.079
Grade 3	0.717 ± 0.196	0.854 ± 0.089

**Table 5:** Class-wise recall (sensitivity) values (five-fold mean ± SD).

Hypertensive Retinopathy Grade	EfficientNetV2-B0 Recall (Mean ± SD)	MobileViT-XXS Recall (Mean ± SD)
Grade 0 (Normal)	0.828 ± 0.065	0.814 ± 0.133
Grade 1 (Mild)	0.358 ± 0.164	0.537 ± 0.180
Grade 2 (Moderate)	0.660 ± 0.227	0.750 ± 0.166
Grade 3 (Severe)	0.664 ± 0.166	0.727 ± 0.096

**Table 6:** Five-fold cross-validation and ensemble performance metrics on the independent test set.

Metric	EfficientNetV2-B0 (5-Fold Avg ± SD)	EfficientNetV2-B0 (Ensemble)	MobileViT-XXS (5-Fold Avg ± SD)	MobileViT-XXS (Ensemble)
Accuracy	0.651 ± 0.075	0.733	0.720 ± 0.035	0.844
Weighted F1-score	0.637 ± 0.076	0.724	0.716 ± 0.035	0.845
Cohen’s Kappa	0.527 ± 0.103	0.638	0.623 ± 0.046	0.791
ROC–AUC (OvR, macro)	0.880 ± 0.026	0.916	0.916 ± 0.009	0.944
Quadratic-Weighted Kappa	0.812 ± 0.069	0.871	0.846 ± 0.030	0.916
Adjacent Accuracy (±1)	0.953 ± 0.025	0.967	0.971 ± 0.006	0.978

**Table 7:** Inference performance on NVIDIA Jetson Orin Nano Developer Kit: 5-fold ensemble (FP32) and single-model and 5-fold-ensemble TensorRT FP16.

Model	Inference Mode	Inference Time	FPS
MobileViT-XXS	Ensemble—Cold Start (1st inference)	6275.7 ms	0.16
MobileViT-XXS	Ensemble—Warm Start (2nd inference)	485.8 ms	2.06
EfficientNetV2-B0	Ensemble—Cold Start (1st inference)	4128.4 ms	0.24
EfficientNetV2-B0	Ensemble—Warm Start (2nd inference)	420.4 ms	2.38
MobileViT-XXS	TensorRT FP16—Single Model	3.76 ± 0.90 ms	265.67
EfficientNetV2-B0	TensorRT FP16—Single Model	3.56 ± 0.91 ms	280.84
MobileViT-XXS	TensorRT FP16—5-fold Ensemble	10.40 ± 1.14 ms	96.19
EfficientNetV2-B0	TensorRT FP16—5-fold Ensemble	11.35 ± 1.09 ms	88.11

**Table 8:** Comparison of the proposed models with representative published studies on automated hypertensive retinopathy classification.

Study	Year	Architecture	Task	Modality	Dataset (Images, *n*)	Grades	Acc (%)	ROC–AUC	Validation
Triwijoyo et al. [8]	2017	Shallow CNN	HR only	Fundus	40 (DRIVE)	Binary	98.6	N/R	Single split
Qureshi et al. [10]	2022	DSC-CNN + LSVM	HR only	Fundus	9500	Binary	95.0	0.96	Single split
Abbas et al. [7]	2023	HDR-EfficientNet-V2	HR + DR	Fundus	36,000+	3-class (Normal, HR, DR)	98.0	0.98	10-fold CV
Bhimavarapu et al. [11]	2024	CNN-SCM + SVM	HR only	Fundus	1200	5-class	98.99	N/R	10-fold CV
Suman et al. [15]	2025	ResNet-50 + ViT hybrid	HR only	Fundus	HRSG (494)	4-class	96.88	N/R	Single split
Şüyun et al. [12]	2025	Triple-stream CNN + HHO	HR only	OCT	1875	4-class (Normal, Stage 1–3)	94.66	N/R	Single split
EfficientNetV2-B0 (Proposed)	2026	EfficientNetV2-B0	HR only	OCT	478	4-class (KWB-insp.)	73.3	0.916	5-fold CV + indep. test
MobileViT-XXS (Proposed)	2026	MobileViT-XXS	HR only	OCT	478	4-class (KWB-insp.)	84.4	0.944	5-fold CV + indep. test

**Table 9:** Qualitative Grad-CAM interpretation across hypertensive retinopathy grades for the illustrated cases of Figure 7 and Figure 8, with the observed attention–pathology concordance and its principal cause.

Model	Grade	Observed Activation Pattern	Attention–Pathology Concordance	Principal Reason for the Observed Behavior
EfficientNetV2-B0	Grade 0	Several discrete high-activation foci over unremarkable background, including regions traversed by horizontal banding	Discordant (no target region exists)	Non-specific texture response; coarse 7 × 7 attribution grid; acquisition artefact not suppressed
EfficientNetV2-B0	Grade 1	Single dominant focus immediately superior to the optic disc; remainder of the field suppressed	Partially concordant	Peripapillary zone in which arteriolar caliber is conventionally assessed; discriminative signal is weak at this grade
EfficientNetV2-B0	Grade 2	Broad response along the inferior vascular arcade plus a focus over a hyporeflective area in the superior field	Partially concordant	Vessel-following response is appropriate to the KWB-inspired criteria; the superior focus is not vessel-related
EfficientNetV2-B0	Grade 3	Extensive activation in the superior field; low response over the lobulated, cyst-like region	Discordant for the dominant finding	Correct grade reached without lesion-level attribution; fold-1 is the weakest of the five models (Table 3)
MobileViT-XXS	Grade 0	Broad, low-to-moderate activation with two moderate foci	Discordant (no target region exists)	Coarse single-layer attribution; non-specific texture response
MobileViT-XXS	Grade 1	Distributed activation with a strong inferior focus and a rim of response adjacent to the optic disc	Partially concordant	Broad distribution is expected of a single coarse attribution map; early-stage signal is weak
MobileViT-XXS	Grade 2	Strong response on the optic disc and along the inferior vessels	Concordant	Vessel- and disc-centered response matching criteria defined on arteriolar caliber and crossings
MobileViT-XXS	Grade 3	Single dominant elongated focus in the superior field; low response over the cyst-like region	Discordant for the dominant finding	No localization supervision; single-layer attribution cannot depict token-level self-attention

Concordance denotes the qualitative spatial agreement between the attention map and the structural findings visible on the corresponding source image; it is not a quantitative localization metric, since no lesion-level reference annotation exists for this dataset (Section 2.8). All maps derive from the fold-1 model of each architecture and are illustrative of a single ensemble member.

## Data Availability

The en-face OCT images are not publicly available owing to institutional restrictions. The patient-level fold assignments, the per-fold training and validation curves, the per-fold confusion matrices, and the evaluation outputs referred to throughout the text are provided in the archived code repository cited in the Code Availability Statement. The source code and pre-trained model weights are archived on Zenodo (DOI: 10.5281/zenodo.21992254).

## Abbreviations

HR: hypertensive retinopathy; OCT: optical coherence tomography; KWB: Keith–Wagener–Barker; CNN: convolutional neural network; ViT: Vision Transformer; ROC–AUC: receiver operating characteristic—area under the curve; OvR: one-vs-rest; QWK: quadratic-weighted kappa; SD: standard deviation; CI: confidence interval; SE: Squeeze-and-Excitation; GAP: global average pooling; Grad-CAM: gradient-weighted class activation mapping; FP32/FP16/INT8: 32-/16-/8-bit floating-point/integer precision; FPS: frames per second; GPU: graphics processing unit; ONNX: Open Neural Network Exchange; DR: diabetic retinopathy; XAI: explainable artificial intelligence.
